# The *KMT2A* recombinome of acute leukemias in 2023

**DOI:** 10.1038/s41375-023-01877-1

**Published:** 2023-04-05

**Authors:** C. Meyer, P. Larghero, B. Almeida Lopes, T. Burmeister, D. Gröger, R. Sutton, N. C. Venn, G. Cazzaniga, L. Corral Abascal, G. Tsaur, L. Fechina, M. Emerenciano, M. S. Pombo-de-Oliveira, T. Lund-Aho, T. Lundán, M. Montonen, V. Juvonen, J. Zuna, J. Trka, P. Ballerini, H. Lapillonne, V. H. J. Van der Velden, E. Sonneveld, E. Delabesse, R. R. C. de Matos, M. L. M. Silva, S. Bomken, K. Katsibardi, M. Keernik, N. Grardel, J. Mason, R. Price, J. Kim, C. Eckert, L. Lo Nigro, C. Bueno, P. Menendez, U. zur Stadt, P. Gameiro, L. Sedék, T. Szczepański, A. Bidet, V. Marcu, K. Shichrur, S. Izraeli, H. O. Madsen, B. W. Schäfer, S. Kubetzko, R. Kim, E. Clappier, H. Trautmann, M. Brüggemann, P. Archer, J. Hancock, J. Alten, A. Möricke, M. Stanulla, J. Lentes, A. K. Bergmann, S. Strehl, S. Köhrer, K. Nebral, M. N. Dworzak, O. A. Haas, C. Arfeuille, A. Caye-Eude, H. Cavé, R. Marschalek

**Affiliations:** 1 DCAL/Institute of Pharm. Biology, Goethe-University, Frankfurt/Main, Germany; 2 Instituto Nacional de Câncer (INCA), Rio de Janeiro, RJ Brazil; 3 Charité – Universitätsmedizin Berlin, corporate member of Freie Universität Berlin and Humboldt-Universität zu Berlin, Dept. of Hematology, Oncology and Tumor Immunology, Berlin, Germany; 4 Molecular Diagnostics, Children’s Cancer Institute, Lowy Cancer Research Centre, UNSW, Sydney, NSW Australia; 5 Tettamanti Research Center, Pediatrics, University of Milano-Bicocca/Fondazione Tettamanti, Monza, Italy; 6 Regional Children’s Hospital, Ekaterinburg, Russian Federation; Research Institute of Medical Cell Technologies, Ekaterinburg, Russian Federation; 7 Laboratory of Clinical Genetics, Fimlab Laboratories, Tampere, Finland; 8 Department of Clinical Chemistry and Laboratory Division, University of Turku and Turku University Hospital, Turku, Finland; 9 CLIP, Department of Paediatric Haematology and Oncology, Second Faculty of Medicine, Charles University and University Hospital Motol, Prague, Czech Republic; 10 Biological Hematology, AP-HP A. Trousseau, Pierre et Marie Curie University, Paris, France; 11 Department of Immunology, Erasmus MC, University Medical Center Rotterdam, Rotterdam, Netherlands; 12 Princess Máxima Center for Pediatric Oncology, Utrecht, Netherlands; 13 Institut Universitaire du Cancer de Toulouse, Toulouse Cedex 9, France; 14 Cytogenetics Department, Bone Marrow Transplantation Unit, National Cancer Institute (INCA), Rio de Janeiro, Brazil; 15 Wolfson Childhood Cancer Research Centre, Translational and Clinical Research Institute, Newcastle University, Newcastle upon Tyne, United Kingdom; 16 Division of Pediatric Hematology-Oncology, First Department of Pediatrics, National and Kapodistrian University of Athens, “Aghia Sophia” Children’s Hospital, Athens, Greece; 17 Genetics and Personalized Medicine Clinic, Tartu University Hospital, Tartu, Estonia; 18 Department of Hematology, CHU Lille, France; 19 Northern Institute for Cancer Research, Newcastle University and the Great North Children’s West Midlands Regional Genetics Laboratory, Birmingham Women’s and Children’s NHS Foundation Trust, Mindelsohn Way, Birmingham, United Kingdom; 20 Department of Laboratory Medicine, Wonju Severance Christian Hospital, Yonsei University Wonju College of Medicine, Wonju, Korea; 21 Charité – Universitätsmedizin Berlin, corporate member of Freie Universität Berlin and Humboldt-Universität zu Berlin, Department of Pediatric Oncology/Hematology, Berlin, Germany; 22 Centro di Riferimento Regionale di Ematologia ed Oncologia Pediatrica, Azienda Policlinico “G. Rodolico”, Catania, Italy; 23 Josep Carreras Leukemia Research Institute. Barcelona, Spanish Network for Advanced Therapies (RICORS-TERAV, ISCIII); Spanish Collaborative Cancer Network (CIBERONC. ISCIII); University of Barcelona, Barcelona, Spain; 24 Josep Carreras Leukemia Research Institute. Barcelona, Spanish Network for Advanced Therapies (RICORS-TERAV, ISCIII); Spanish Collaborative Cancer Network (CIBERONC. ISCIII); Department of Biomedicine. University of Barcelona; and Institució Catalana de Recerca i Estudis Avançats (ICREA), Barcelona, Spain; 25 Pediatric Hematology and Oncology and CoALL Study Center, University Medical Center Hamburg-Eppendorf, Hamburg, Germany; 26 Instituto Português de Oncologia, Departament of Hematology, Lisbon, Portugal; 27 Department of Pediatric Hematology and Oncology, Medical University of Silesia, Zabrze, Poland; 28 Laboratoire d’Hématologie Biologique, CHU Bordeaux, Bordeaux, France; 29 Hematology Laboratory, Sheba Medical Center, Tel-Hashomer, Israel; 30 Molecular Oncology Laboratory, Schneider Children’s Medical Center of Israel, Petah Tikva, Israel; 31 Pediatric Hematology-Oncology, Schneider Children’s Medical Center, Petah Tikva, and Sackler Faculty of Medicine, Tel Aviv University, Tel Aviv, Israel; 32 Department of Clinical Immunology, Copenhagen University Hospital Rigshospitalet, Copenhagen, Denmark; 33 Division of Oncology and Children’s Research Centre, University Children’s Hospital Zurich, Zurich, Switzerland; 34 Hematology Laboratory, Saint Louis Hospital, Assistance Publique-Hôpitaux de Paris (AP-HP), Paris, France; 35 Université Paris Cité, INSERM/CNRS U944/UMR7212, Institut de recherche Saint-Louis, Paris, France; 36 Laboratory for Specialized Hematological Diagnostics, Medical Department II, University Hospital Schleswig-Holstein, Kiel, Germany; 37 Bristol Genetics Laboratory, North Bristol NHS Trust, Bristol, United Kingdom; 38 Department of Pediatrics, University Hospital Schleswig-Holstein, Kiel, Germany; 39 Department of Pediatrics, MHH, Hanover, Germany; 40 Institute of Human Genetics, Medical School Hannover, Hannover, Germany; 41 St. Anna Children’s Cancer Research Institute (CCRI), Vienna, Austria; 42 Labdia Labordiagnostik, Vienna, Austria; 43 St. Anna Children’s Hospital, Medical University of Vienna, Vienna, Austria; 44 Genetics Department, AP-HP, Hopital Robert Debré, Paris, France; 45 Université Paris Cité, Inserm U1131, Institut de recherche Saint-Louis, Paris, France

**Keywords:** Genetics research, Cancer genetics

## Abstract

Chromosomal rearrangements of the human *KMT2A/MLL* gene are associated with *de novo* as well as therapy-induced infant, pediatric, and adult acute leukemias. Here, we present the data obtained from 3401 acute leukemia patients that have been analyzed between 2003 and 2022. Genomic breakpoints within the *KMT2A* gene and the involved translocation partner genes (TPGs) and *KMT2A*-partial tandem duplications (PTDs) were determined. Including the published data from the literature, a total of 107 in-frame *KMT2A* gene fusions have been identified so far. Further 16 rearrangements were out-of-frame fusions, 18 patients had no partner gene fused to 5’-*KMT2A*, two patients had a 5’-*KMT2A* deletion, and one *ETV6::RUNX1* patient had an *KMT2A* insertion at the breakpoint. The seven most frequent TPGs and PTDs account for more than 90% of all recombinations of the *KMT2A*, 37 occur recurrently and 63 were identified so far only once. This study provides a comprehensive analysis of the *KMT2A* recombinome in acute leukemia patients. Besides the scientific gain of information, genomic breakpoint sequences of these patients were used to monitor minimal residual disease (MRD). Thus, this work may be directly translated from the bench to the bedside of patients and meet the clinical needs to improve patient survival.

## Introduction

Chromosomal rearrangements involving the human *KMT2A* gene (NM_001412597.1) are recurrently associated with the disease phenotype of acute leukemias [1, 2]. The presence of distinct *KMT2A* rearrangements is an independent dismal prognostic factor, while very few *KMT2A* rearrangements confer either a good or intermediate outcome [3, 4]. It became also clear from recent studies that the follow-up of patients during their treatment and therapy-adjustments based on individual MRD monitoring has a very strong impact on outcome [5–7]. For this purpose, we established more than 20 years ago a diagnostic network that allowed different study groups and clinical centers to obtain genomic *KMT2A* breakpoint sequences that can be directly used for quantifying MRD levels in their patients. The current workflow to identify *KMT2A* rearrangements includes still a pre-screening step (cytogenetic analyses [8, 9], split-signal fluorescence in situ hybridization (FISH) [10–12], RT-PCR [13] or RNA-Seq) at study/diagnostic centers. Pre-screened samples derived from infant, pediatric, and adult leukemia patients were then sent for analysis to the Frankfurt Diagnostic Center of Acute Leukemia (DCAL). These patient samples were then analyzed by a combination of long-distance inverse PCR (LDI-PCR) [14], LD multiplex PCR, and by targeted sequencing of full-length *KMT2A* by next-generation sequencing (NGS) [15, 16]. This allowed us to identify reciprocal translocations, complex chromosomal rearrangements, gene internal duplications, deletions or inversions on chromosome 11, and *KMT2A* gene insertions into other chromosomes, or vice versa, the insertion of partner chromosome material into the *KMT2A* gene located at 11q23.3. As a result, at least one patient-specific *KMT2A* fusion sequence was obtained and used for establishing patient-specific qPCR assays to monitor MRD of the patient in the clinical setting. It also allowed us to identify unknown fusion partner genes. The results of this effort will be presented, statistically analyzed and discussed.

## Methods

### Patient material

Genomic DNA was isolated from bone marrow and/or peripheral blood samples of leukemia patients and sent to DCAL. Patient samples were obtained from different diagnostic centers worldwide involved in different study groups (Australia, Austria, Brazil, Czech Republic, Denmark, Estonia, Finland, France, Germany, Greece, Israel, Italy, Netherlands, Poland, Portugal, Slovakia, Spain, Russian Federation, Switzerland, United Kingdom) Informed consent was obtained from all patients or patients’ parents/legal guardians and control individuals.

### Detection of chromosomal breakpoints by LDI-PCR, LD multiplex PCR, and targeted NGS

For LDI-PCR all DNA samples were treated and analyzed as described [17–20]. Briefly, genomic patient DNA was digested with restriction enzymes and re-ligated to form DNA circles prior to LDI-PCR analyses. Restriction polymorphic PCR amplimers were isolated from the gel and subjected to DNA sequence analyses. The most frequent TPGs were in general analyzed by LD multiplex PCR. Alternatively genomic patient DNA was subjected to targeted NGS, as previously described [15, 16]. For three patients the fusion sites were obtained by either RNA-Seq (*n* = 2) or RT-PCR experiments (*n* = 1). Idiosyncratic genomic DNA fusion sequences were made available to the sender of the DNA sample for patient-specific MRD surveillance.

### Data evaluation and statistical analyses

All clinical and experimental patient data were implemented into a database program (FileMaker Pro) for further analysis. Information about all individual patients was used to compare all defined subgroups and to perform statistical analyses. Chi-Square distribution analyses were performed by using the following website: www.mathsisfun.com/data//chi-square-calculator.html. The database offers the possibility to analyze specific patient cohorts and we will share these data with requesting scientists.

### Nomenclature

We use the current HUGO nomenclature (https://www.genenames.org/) throughout the text. Some TPGs are listed below with former gene names: *KMT2A* = former *MLL, ALL1, HRX; AFF1* = former *AF4; AFF3* = former *LAF4; AFF4* = former *AF5; MLLT1* = former *ENL; MLLT3* = former *AF9*; *AFDN* = former *AF6*; *MLLT6* = former *AF17*; *MLLT10* = former *AF10; MLLT11* = former *AF1Q*.

## Results

### The study cohort

**Fig. 1:**
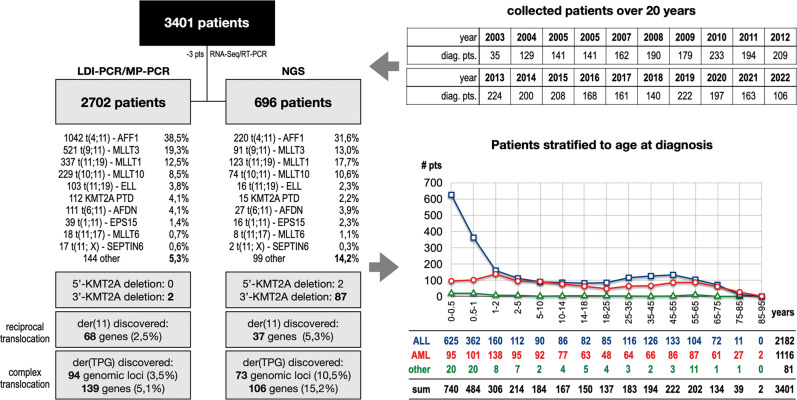
Overview of all analyzed patients. In the last 20 years a total of 3401 acute leukemia patients have been analyzed at the DCAL. Most of these patients were analyzed by the described LDI-PCR technology (2702 patients), a technique that has been successfully substituted by targeted NGS over the last years (696 pts). The benefit of using NGS over LDI-PCR (see left part) is clearly shown by the differences in novel target gene identification (15.2% vs. 5.1%), as well as the identification of 5’- and 3’-*KMT2A* deletions, which is per se impossible by using PCR-based techniques. The age stratification of all investigated patients is shown on the right bottom, clearly indicating that patients with ALL below 1 year of age at diagnosis represent a unique patient population with a high incidence for *KMT2A* rearrangements.

To analyze the recombinome of the human *KMT2A* gene, pre-screened and not-prescreened acute leukemia samples were obtained between 2003 and 2022. As described in Methods, patient genomic DNA was either analyzed by PCR or targeted NGS to obtain the genetic information of rearranged *KMT2A* fusion alleles. For most of the investigated cases, we obtained all clinical information (gender, age at diagnosis, disease type and subtype or information about *de novo* or secondary leukemia), which is necessary for subsequent data processing. Patients for which we were unable to obtain the relevant clinical data, or which were *KMT2A*-r negative in our analyses were excluded from the present study. The results of the remaining 3401 patients are summarized in Fig. 1 and Table 1.

**Table 1:** Overview about all investigated direct KMT2A fusion genes or KMT2A deviations.

#	direct TPG	Infant			Pediatric			Adult			not age classifed			Total
		ALL	AML	other	ALL	AML	other	ALL	AML	other	ALL	AML	other	
1	AFF1	477	3	12	215	1	6	535	5	0	6	0	2	1262
2	MLLT3	157	56	5	91	170	3	8	108	3	2	5	4	612
3	MLLT1	234	2	6	100	28	1	66	16	2	4	1	0	460
4	MLLT10	59	54	8	17	109	4	3	44	2	1	2	1	304
5	AFDN	3	3	0	31	42	0	10	45	1	2	1	0	138
6	KMT2A-PTD	0	0	0	0	7	0	1	113	6	0	0	0	127
7	ELL	0	30	1	0	32	1	1	51	3	0	0	0	119
8	EPS15	22	2	3	8	5	0	4	7	1	2	1	0	55
9	USP2	3	0	4	17	1	4	1	0	0	0	0	0	30
10	MLLT11	1	13	0	0	10	0	0	4	0	0	0	0	28
11	MLLT6	5	0	0	3	5	0	0	12	0	0	1	1	27
12	SEPTIN6	0	7	0	0	10	0	0	2	0	0	0	0	19
13	SEPTIN9	0	2	0	0	6	0	0	6	0	0	0	0	14
14	AFF3	4	0	0	7	0	0	0	0	0	0	0	0	11
15	CBL	0	0	0	7	0	0	1	1	1	0	0	0	10
16	SEPTIN5	0	2	0	1	3	0	0	2	0	0	0	0	8
17	ABI1	0	3	0	0	5	0	0	0	0	0	0	0	8
18	CREBBP	0	0	0	1	2	2	0	0	0	0	0	1	6
19	KNL1	0	0	0	1	3	0	0	1	1	0	0	0	6
20	TET1	0	0	0	0	1	0	2	3	0	0	0	0	6
21	TNRC18	1	0	0	5	0	0	0	0	0	0	0	0	6
22	MYO1F	0	4	0	0	1	0	0	0	0	0	0	0	5
23	MAML2	0	0	0	2	0	0	1	0	1	0	0	0	4
24	PICALM	1	1	0	1	0	0	0	1	0	0	0	0	4
25	FLNA	0	2	0	0	2	0	0	0	0	0	0	0	4
26	ARHGEF12	0	0	0	0	1	0	0	1	1	0	0	0	3
27	CLTC	2	0	0	0	1	0	0	0	0	0	0	0	3
28	GAS7	0	1	0	1	0	0	0	0	0	0	0	1	3
29	NEBL	0	1	0	0	2	0	0	0	0	0	0	0	3
30	PRPF19	1	0	0	2	0	0	0	0	0	0	0	0	3
31	SEPTIN11	0	0	0	1	1	0	1	0	0	0	0	0	3
32	USP8	0	0	0	2	0	1	0	0	0	0	0	0	3
33	ACTN4	0	0	0	0	1	0	1	0	0	0	0	0	2
34	AFF4	2	0	0	0	0	0	0	0	0	0	0	0	2
35	BTBD18	2	0	0	0	0	0	0	0	0	0	0	0	2
36	CEP170B	0	0	0	0	0	0	0	2	0	0	0	0	2
37	CIP2A	0	2	0	0	0	0	0	0	0	0	0	0	2
38	DCP1A	1	0	0	0	1	0	0	0	0	0	0	0	2
39	FOXO3	0	0	0	2	0	0	0	0	0	0	0	0	2
40	ABI2	0	2	0	0	0	0	0	0	0	0	0	0	2
41	ACACA	0	0	0	0	1	0	0	0	0	0	0	0	1
42	ACNT2	1	0	0	0	0	0	0	0	0	0	0	0	1
43	AKAP13	0	0	0	0	0	0	0	1	0	0	0	0	1
44	ARHGAP26	0	0	0	0	1	0	0	0	0	0	0	0	1
45	ARHGEF17	0	0	0	0	1	0	0	0	0	0	0	0	1
46	BCAS4	0	0	0	0	0	0	1	0	0	0	0	0	1
47	C2CD3	0	0	0	0	0	0	0	1	0	0	0	0	1
48	CASP8AP2	0	0	0	0	0	0	0	1	0	0	0	0	1
49	CLIP2	0	0	0	1	0	0	0	0	0	0	0	0	1
50	CLTA	1	0	0	0	0	0	0	0	0	0	0	0	1
51	CT45S2	0	0	0	0	0	1	0	0	0	0	0	0	1
52	DCPS	0	0	0	0	0	0	0	1	0	0	0	0	1
53	FAM13A	0	0	0	0	0	0	1	0	0	0	0	0	1
54	FNBP1	0	1	0	0	0	0	0	0	0	0	0	0	1
55	GIGYF2	0	0	0	1	0	0	0	0	0	0	0	0	1
56	GMPS	0	0	0	0	0	0	0	1	0	0	0	0	1
57	ITSN1	0	1	0	0	0	0	0	0	0	0	0	0	1
58	KIF2A	0	0	0	0	0	0	1	0	0	0	0	0	1
59	LAMC3	0	0	0	0	1	0	0	0	0	0	0	0	1
60	LASP1	0	0	0	0	1	0	0	0	0	0	0	0	1
61	MATR3	0	0	0	0	0	0	1	0	0	0	0	0	1
62	ME2	0	0	0	0	0	0	0	1	0	0	0	0	1
63	MRTFA	0	0	0	1	0	0	0	0	0	0	0	0	1
64	MYH11	0	0	0	0	0	0	0	1	0	0	0	0	1
65	NRIP3	0	1	0	0	0	0	0	0	0	0	0	0	1
66	PDS5A	0	0	0	0	0	0	0	1	0	0	0	0	1
67	PFDN4	0	0	0	1	0	0	0	0	0	0	0	0	1
68	PRRC1	0	0	0	0	0	0	1	0	0	0	0	0	1
69	RABGAP1	0	0	0	0	0	1	0	0	0	0	0	0	1
70	RANBP3	0	0	0	1	0	0	0	0	0	0	0	0	1
71	RUNDC3B	0	0	0	1	0	0	0	0	0	0	0	0	1
72	SEPTIN2	0	0	0	0	1	0	0	0	0	0	0	0	1
73	SEPTIN3	0	0	0	0	0	0	0	1	0	0	0	0	1
74	SMAP1	0	0	0	0	0	0	0	1	0	0	0	0	1
75	SNX9	0	0	0	0	1	0	0	0	0	0	0	0	1
76	STK4	0	0	0	1	0	0	0	0	0	0	0	0	1
77	TCF12	0	0	0	0	0	0	0	1	0	0	0	0	1
78	TOP3A	0	0	0	0	0	0	0	1	0	0	0	0	1
79	VAV1	0	0	0	0	1	0	0	0	0	0	0	0	1
80	**AP2A2**	0	0	0	0	0	0	0	1	0	0	0	0	1
81	**ARHGAP32**	0	0	0	0	0	0	1	0	0	0	0	0	1
82	**BCL9L**	0	0	0	1	0	0	0	0	0	0	0	0	1
83	**BUD13**	0	0	0	0	1	0	0	0	0	0	0	0	1
84	**CEP164**	0	0	0	0	0	0	1	0	0	0	0	0	1
85	**DAPK1**	0	0	0	0	0	0	0	0	0	0	1	0	1
86	**DDX6**	0	0	0	1	0	0	0	0	0	0	0	0	1
87	**EEFSEC**	1	0	0	0	0	0	0	0	0	0	0	0	1
88	**LOC100131626**	0	0	0	0	0	0	0	0	1	0	0	0	1
89	**MGMT**	0	0	0	1	0	0	0	0	0	0	0	0	1
90	**MIDN**	1	0	0	0	0	0	0	0	0	0	0	0	1
91	**NOX4**	0	0	0	0	0	0	0	1	0	0	0	0	1
92	**NUP153**	0	0	0	1	0	0	0	0	0	0	0	0	1
93	**OPCML**	0	0	0	0	0	0	1	0	0	0	0	0	1
94	**RANBP3**-**DT**	0	0	0	0	0	0	0	0	0	0	1	0	1
95	no der(11) / CPX	5	3	1	0	3	0	0	1	0	1	0	2	16
96	der(11) / no TPG	3	1	0	3	2	1	4	2	2	0	0	0	18
97	5’-KMT2A del	0	0	0	1	1	0	0	0	0	0	0	0	2
98	RUNX1-ETV6 integr.	0	0	0	0	0	1	0	0	0	0	0	0	1
	SUM	987	197	40	530	465	26	647	441	25	18	13	12	3401

All fusion genes that have been analyzed at the DCAL and their distribution between infant, pediatric and adult leukemia patients is shown. Total numbers are given for each patient group separated in ALL, AML and other (diseases). The 11 most frequent direct TPGs and *KMT2A*-PTDs were separated from other 29 fusion genes that were recurrently diagnosed. Additional 54 TPGs were identified so far only once, of which 15 were out-of-frame fusions (marked in bold). We also identified 16 *KMT2A* rearrangements with no direct *KMT2A* fusion gene, but in some cases with a reciprocal *KMT2A* fusion (#95), and additional 18 patients had no partner gene fused to 5’-*KMT2A* (#96). Two cases were identified with a deletion of 5’-*KMT2A* (#97), and one patient had an *ETV6-RUNX1* fusion gene with a *KMT2A* insertion at the breakpoint (#98).

### Age distribution according to clinical subtypes

The cohort was first analyzed according to the age at diagnosis. As displayed in Fig. 1, the age distribution is quite similar to the expected distributions known from other cancer registries, where infant ALL has always a very high incidence in the first 2 years of life. Here, the high infant *KMT2A*-r ALL incidence peaks within the first year of life with about 48% *AFF1*, 24% *MLLT1* and 16% *MLLT3* cases. Then it declines during the pediatric and young adult phase and increases slightly until 55 years, and finally declines due to dropping patient numbers. A similar picture was observed with *KMT2A*-r AML patients, however, lacking the postnatal peak seen for infant ALL. In this study, the cohort was separated by fusion partner gene and the following age groups (see Table 1): 1. “infant acute leukemia” (≤12 months; *n* = 1224: 987 ALL, 197 AML, 40 other), 2. “pediatric acute leukemia” (>12 months–18 years; *n* = 1021: 530 ALL, 465 AML, 26 other), 3. “adult acute leukemia patient” (>18 years; *n* = 1113: 647 ALL, 441 AML, 25 other), 4. “age nonannotated patients” (*n* = 43: 18 ALL, 13 AML, 12 other). In Suppl. Table S1, patients exhibiting the 12 most abundant fusion partner genes were categorized according to the number of patients, mean age at diagnosis, gender (female *n* = 1783: 656 infant, 491 pediatric, 633 adult, 3 NA; male n = 1561: 560 infant, 528 pediatric, 470 adult, 3 NA), patient subgroups (infant/pediatric/adult (I/P/A), therapy-induced leukemia (TIL), complex leukemic rearrangements (CL) and major breakpoint distribution. Due to missing clinical information, 57 patients had no gender information available (8 infant, 2 pediatric, 10 adult, 37 NA), and for 43 patients no age classification could be performed.

### Identification of *KMT2A* rearrangements and their distribution in clinical subgroups

**Fig. 2:**
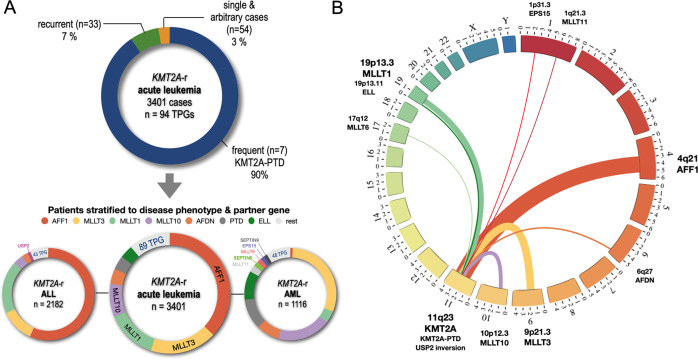
Classification of patients according to TPG and disease phenotype. **A** The 7 most frequent *KMT2A* fusion partners (*AFF1, MLLT3, MLLT1, MLLT10, AFDN, KMT2A -*PTDs and *ELL*) represented more than 90% of the investigated patients. The next 33 recurrently diagnosed fusion partners represented 7% of cases, while all 54 unique fusion partners represent only 3% of the cohort (*n* = 3401). This patient cohort was divided into 2182 ALL (left) and 1116 AML patients (right). The 7 most frequently diagnosed fusion partners are color coded as indicated on top of the circular plots. Noteworthy, *AFF1*, *MLLT1* and *MLLT3* represented 87% of all diagnosed ALL patients. *KMT2A::USP2* fusion were solely diagnosed in the ALL patient group and are indicated separately. Within the AML group, the fusion partners *MLLT3, MLLT1, MLLT10, AFDN, KMT2A-*PTDs *and ELL* cases accounted for 82% of diagnosed patients. Genes like *MLLT11*, *SEPTIN6*, *MLLT6*, *EPS15* and *SEPTIN* 9 account for additional 8.3% of cases and are indicated separately. **B** Circos plot for the 11 most frequent *KMT2A* fusion partner genes: *EPS15, MLLT11, AFF1, AFDN, MLLT3, MLLT10, KMT2A-*PTDs*, USP2, MLLT6, ELL* and *MLLT1* (sorted according to their chromosomal order).

The most frequent *KMT2A* rearrangements in the two disease subgroups ALL and AML are summarized in Fig. 2A (left side). ALL patients (*n* = 2182) displayed the following rearrangements: *AFF1* (*n* = 1233; 56.5%), *MLLT1* (*n* = 404; 18.5%), *MLLT3* (*n* = 258; 11.8%), *MLLT10* gene (*n* = 80; 3.7%), *AFDN* (46; 2.1%), *EPS15* (*n* = 36; 1.6%), *USP2* (*n* = *21;* 0.9%), *ELL* (*n* = 1; 0.05%), *KMT2A-*PTD (*n* = 1; 0.05%), and 43 other *KMT2A* rearrangements (*ACTN2, ACNT4, 11x AFF3, 2x AFF4, ARHGAP32, BCAS4, BCL9L*, 2x *BTBD18*, 8x *CBL, CEP164, CLIP2, CLTA*, 2x *CLTC, CREBBP, DCP1A, DDX6, EEFSEC, FAM13A*, 2x *FOXO3, GAS7, GIGYT2, KIF2A, KNL1*, 3x *MAML2, MATR3, MGMT, MIDN, MLLT11, MRTFA, NUP153, OPCML, PFDN4*, 2x *PICALM*, 3x *PRPF19, PRRC1, RANBP3, RUNDC3B, SEPTIN5*, 2x *SEPTIN11, STK4*, 2x *TET1*, 6x *TRNC18*, 2x *USP8* and 17 patients with no direct fusion gene).

AML patients (*n* = 1116) displayed a more diverse number of partner genes: *MLLT3* (*n* = 339; 30.4%), *MLLT10* (*n* = 209; 18.7%), *KMT2A*-PTDs (*n* = 120; 10.7%), *ELL* (*n* = 113; 10.1%), *AFDN* (*n* = 91; 8.1%), *MLLT1* (*n* = 47; 4.2%), *MLLT11* (*n* = 27; 2.4%), *SEPTIN6* (*n* = 19; 1.7%), *MLLT6* (*n* = 18; 1.6%), *EPS15* (*n* = 15; 1.3%), *SEPTIN9* (*n* = 14; 1.3%), *AFF1* (*n* = 9; 0.8%), and 48 other *KMT2A* rearrangements (8x *ABI1*, 2x *ABI2, ACACA, ACTN4, AKAP13, AP2A2, ARHGAP26*, 2x *ARHGEF12, ARHGEF17, BUD13, C2CD3, CASP8AP2, CBL*, 2x *CEP170B*, 2x *CIP2A, CLTC, 2x CREBBP, DAPK1, DCPS, 4x FLNA, FNBP1, GAS7, GMPS, ITF46, ITSN1*, 4x *KNL1, LAMC3, LASP1, ME2, MYH11*, 5x *MYOF1*, 3x *NEBL, NOX4, NRIP3, PDS5A*, 2x *PICALM, RANBP3-DT, SEPTIN2, SEPTIN3*, 7x *SEPTIN5, SEPTIN11, SMAP, SNX9, TCF12*, 4x *TET1, TOP3A, USP2, VAV1* and 12 patients with no direct fusion gene).

**Fig. 3:**
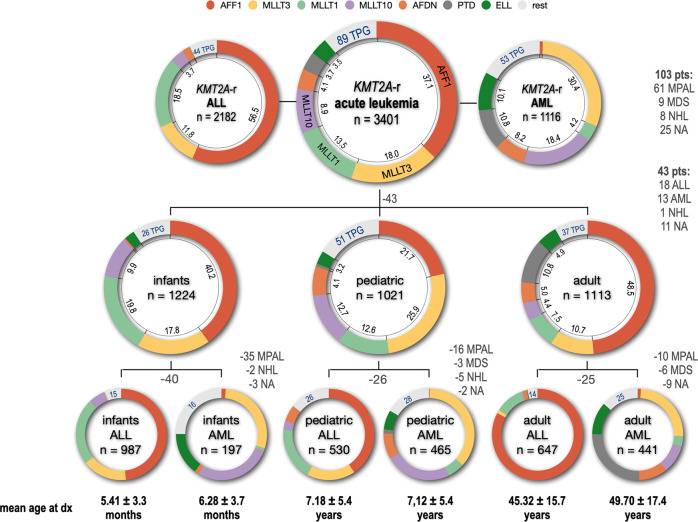
Classification of all fusion partner genes by disease phenotype and age classification. All 3401 diagnosed patients were grouped by their diagnosed disease type (ALL: 2182; AML: 1116; 103 pts had other diseases listed on the right). Since we had for 43 pts no age information at diagnosis, they were excluded from being further subdivided into the age groups infant (*n* = 1224), pediatric (*n* = 1021) and adult patients (*n* = 1113). All 3 age groups were again subdivided in ALL or AML subgroups (infant ALL = 987 pts; infant AML = 197 pts; pediatric ALL = 530 pts; pediatric AML = 465 pts; adult ALL = 647 pts; adult AML = 441 pts). Number of patients with missing information or different disease subtypes are indicated (grey letters). The mean age for all 6 subgroups is given below, either in months or years ± SD. The distribution of the 7 most frequent fusion partners is given by different colors (color code on top) and their frequency in percent. The additional number of identified fusion partner genes are given by blue numbers for each subgroup.

The distribution of the 11 most frequent *KMT2A* rearrangements (*EPS15, MLLT11, AFF1, AFDN, MLLT3, MLLT10, USP2, KMT2A-*PTD*, MLLT6, ELL, MLLT1* and *SEPTIN6*) is displayed in a circos plot in Fig. 2B (right side). Figure 3 summarizes the distribution according to age at diagnosis (I/P/A) for the 7 most frequent fusion partner genes and their corresponding disease phenotype taken from Table 1 (ALL and AML).

Infant ALL patients (*n* = 987) displayed the following rearrangements: *AFF1* (*n* = 477; 48,3%), *MLLT1* (*n* = 234; 24%) *MLLT3* (*n* = 157; 16%), *MLLT10* (*n* = 59; 6%), *EPS15* (*n* = 22; 2,2%) *AFDN* (*n* = 3; 0,3%), and 15 other *KMT2A* rearrangements. Infant AML patients (*n* = 197) displayed the following rearrangements: *MLLT3* (*n* = 56; 28.4%), *MLLT10* (*n* = 54; 27.4%), *ELL* (*n* = 30; 15.2%), *AFDN* (*n* = 3; 1.5%), *AFF1* (*n* = 3; 1.5%), *EPS15* (*n* = 2; 1.0%), *MLLT1* (*n* = 2; 1.0%) and 16 other *KMT2A* rearrangements. Pediatric ALL patients (*n* = 530) displayed the following rearrangements: *AFF1* (*n* = 215; 40.6%), *MLLT1* (*n* = 100; 18.9%), *MLLT3* (*n* = 91; 17.2%), *AFDN* (*n* = 31; 5.8%), *MLLT10* (*n* = 17; 3.2%), *EPS15* (*n* = 8; 1.5%) and 26 other *KMT2A* rearrangements. Pediatric AML patients (*n* = 465) displayed the following rearrangements: *MLLT3* (*n* = 170; 36.6%), *ELL* (*n* = 32; 6.7%), *MLLT10* (*n* = 109; 23.4%), *AFDN* (*n* = 42; 9.0%), *MLLT1* (*n* = 28; 6.0%), *KMT2A*-PTD (*n* = 7; 1.5%), *EPS15* (*n* = 5; 1.1%), *AFF1* (*n* = 1; 0.2%) and 28 other *KMT2A* rearrangements. Adult ALL patients (*n* = 647) displayed the following rearrangements: *AFF1* (*n* = 535; 82.7%), *MLLT1* (*n* = 66; 10.2%), *AFDN* (*n* = 10; 1.5%), *MLLT3* (*n* = 8; 1.2%), *EPS15* (*n* = 4; 0.6%), *MLLT10* (*n* = 3; 0.5%), *ELL* (*n* = 1; 0.1%), *KMT2A*-PTD (*n* = 1; 0.1%) and 14 other *KMT2A* rearrangements. Adult AML patients (*n* = 441) displayed the following rearrangements: *KMT2A*-PTD (*n* = 113; 25.6%), *MLLT3* (*n* = 108; 24.5%), *ELL* (*n* = 51; 11.6%), *AFDN* (*n* = 45; 10.2%), *MLLT10* (*n* = 44; 10.0%), *MLLT1* (*n* = 16; 3.6%), *EPS15* (*n* = 7; 1.6%), *AFF1* (*n* = 5; 1.1%) and 25 other *KMT2A* rearrangements.

Based on the above distribution, about 94.3% of all ALL patients (*n* = 2182) were characterized by 6 fusion genes *KMT2A::AFF1*, *KMT2A::MLLT1*, *KMT2A::MLLT3*, *KMT2A::MLLT10*, *KMT2A::AFDN and KMT2A::EPS15*. About 84.8% of all AML patients (*n* = 1116) were characterized by 7 fusion genes *KMT2A::MLLT3*, *KMT2A::MLLT10*, *KMT2A*-PTD*, KMT2A::ELL*, *KMT2A::AFDN*, *KMT2A::MLLT1*, and *KMT2A::MLLT11*.

These results are in line with previously published data about the frequency and distribution of different *KMT2A* fusion partner genes [21, 22]. This updated information is highly relevant for diagnostic purposes and the establishment of RT-PCR-based multiplex screening methods [13].

### Breakpoint distribution according to clinical subtypes

**Fig. 4:**
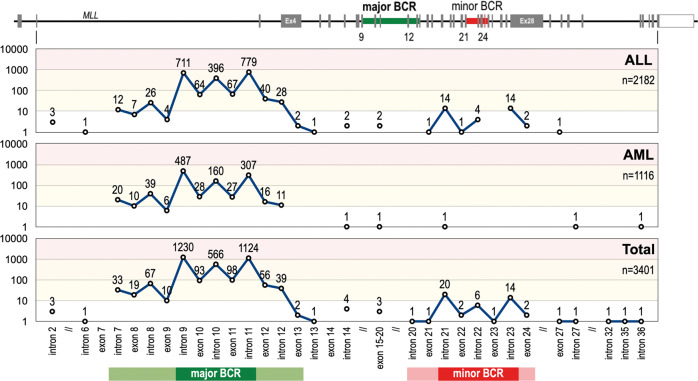
Breakpoint distribution within the *KMT2A* gene. *Top:* The *KMT2A* gene with its 37 exons gene structure (NM_001412597.1) The major and minor BCR are indicated by green and red areas. Below: the number of breakpoint starting from intron 2 until intron 36 is displayed in a logarithmic scale for the disease subgroups ALL (*n* = 2182), AML (*n* = 1116) and the total analyzed patients (*n* = 3401). From this analysis it became clear that breakpoints in the minor BCR of *KMT2A* is a ALL-specific feature, which is nearly absent (only 1 patient) in AML patients. Noteworthy, the 4 breakpoints upstream of the major BCR were associated with ALL, the interim breakpoints (between intron 12 and exon 20) with ALL, AML and MPAL, while the breakpoints downstream of the minor BCR were associated with ALL, AML, MPAL and NHL. the most prominent areas for major and minor BCR are indicated by darker colors (major BCR is intron 9 - intron 11; minor BCR is intron 21 - intron 23).

We also investigated also the breakpoint distribution of the *KMT2A* recombinome. The major breakpoint cluster region (BCR1) can be mapped between *KMT2A* intron 7 and *KMT2A* exon 13, and the minor BCR (BCR2) between intron 20 and exon 24. The majority of patients (*n* = 3336; 98%) showed breakpoints in BCR1 while a minority (*n* = 47; 1,4%) was found in BCR2. When restricting this distribution analysis only to our NGS data, then the distribution between BCR1 and BCR2 is 94% vs. 6%, respectively. The remaining breakpoints (*n* = 17; 0,6%) were found up-stream of BCR1 (*n* = 4), between BCR1 and BCR2 (*n* = 8), and downstream of BCR2 (*n* = 5) (see Fig. 4). We also analyzed the data according to the leukemia phenotype AML or ALL. While ALL breakpoints are found in BCR1 and BCR2, breakpoints in AML patients nearly exclusively occur in BCR1.

The distribution of the 13 most important TPGs and PTDs (*AFF1, MLLT3, MLLT1, MLLT10, ELL, KMT2A*-PTD, *AFDN, EPS15, USP2, MLLT11, MLLT6, SEPTIN6, SEPTIN9, and all others*) are summarized in Suppl. Table S2. The Table also contains information about gender, age, disease classification, TIL, and complex leukemia (CL) cases regarding their breakpoint distribution. Excluded from Suppl. Table S2 were again patients with no gender information *(n* = 57) or no age information at diagnosis (*n* = 43)

It has recently been demonstrated that the localization of breakpoints, particularly within the major BCR, has an impact on cancer biology and clinical behavior: breakpoints within *KMT2A* intron 11 are associated with poorer outcome [23]. Therefore, we first compiled the breakpoint distribution for all 3401 patients. Specific features of *KMT2A* intron 9 (4 Alu repetitive elements of which 3 are transcriptionally active) and *KMT2A* intron 11 (sensitivity against cytotoxic drugs, a DNase1 hypersensitive site [24], an apoptotic cleavage site [25], an RNA Polymerase II binding site [26] and Topoisomerase II binding sites [27]) may account for an increase of DNA double-strand breaks due to these molecular features. As shown in Suppl. Table S2, deviations from the mean distribution in the major BCR were observed for *MLLT1, KMT2A*-PTD*, AFDN, MLLT11, SEPTIN6, SEPTIN9 and MLLT6*. The fusion partner genes *MLLT1* and *SEPTIN6* had preferentially *KMT2A* intron 11 breaks, while all others tended to bear *KMT2A* intron 9 or upstream recombination events, e.g. *SEPTIN9* where breakpoints are shifting to regions even upstream of intron 9 because of the intron phase of the BCR of this partner gene. Of interest, also therapy-induced acute leukemias shifted significantly to *KMT2A* intron 11 breakpoints. None of the other parameters (gender, age classes at diagnosis or diseases subtype) displayed a significant variation from the overall breakpoint distribution.

For a more detailed analysis, we subdivided the *KMT2A* BCR1 into three subregions: (A) exon 9 - intron 9 = 1761 bp; (B) exon 10 - intron 10 = 679 bp; (C) exon 11 - intron 11 - exon 12 - intron 12 and exon 13 = 5026 bp. The functional cut is between regions A-B and C (separating the regions from exon 9 until intron 10 from the region of exon 11 to exon 13). The observed ‘mean distribution’ (MD) for these three *KMT2A* breakpoint regions was A = 37.2%, B = 19.8% and C = 39.5% for all 3401 patients as listed in Suppl. Table S2. We decided not to use a ‘random distribution model’ (RDM) of chromosomal breakpoints, because this is only based on the length of each DNA region, which does not take into account the above-mentioned molecular features. In these subsequent analyses, all patients were investigated for their fusion partner gene in correlation with age class at diagnosis (I/P/A), gender, TIL, CL, disease subtypes and the precise breakpoint distribution.

A more detailed analyses (Suppl. Table S3) showed that more fusion partners diverged from the mean deviation and revealed 10 subgroups with breakpoints in *KMT2A* exons 11–13, and 25 subgroups with preference for *KMT2A* exon 9 to intron 10 (all marked in orange). This finding clearly argues that certain fusion genes have a selective preference for distinct breakpoints, most likely because of specific functions of the respective fusion proteins. As an example, infant *KMT2A::AFF1* patients show breakpoints predominantly localizing to *KMT2A* intron 11, while adult patients displayed a shift to *KMT2A* intron 9 and intron 10. *KMT2A::MLLT10* patients of the pediatric group display a shift towards *KMT2A* intron 9. *KMT2A::ELL* patients show the opposite of *KMT2A::AFF1* patients, namely that pediatric patients have a preference for *KMT2A* intron 9 breakpoints, while pediatric and adult patients have a clear preference for *KMT2A* intron 11. In *KMT2A::AFDN* patients the breakpoints are mostly occurring in *KMT2A* intron 9. Similar observations were made for the rarer fusion partner *MLLT11* (significantly shifting towards *KMT2A* introns 9 and 10), *MLLT6* (significantly towards *KMT2A* introns 8 and 9), *EPS15* (significantly towards *KMT2A* intron 11 in adult patients), *SEPTIN6* (significantly shifting towards *KMT2A* intron 11 in pediatric and adult patients) and *SEPTIN9* (significantly shifting towards *KMT2A* introns 7–9). Noteworthy, the observed shift of *KMT2A* breakpoints towards intron 11 in the adult patient group with *MLLT3* fusions was clearly linked to therapy-induced leukemia. This was not the case for *ELL*, *EPS15* or *SEPTIN6* fusions. Whether these findings have an impact on clinical outcome is yet unclear, but it has been recently shown that breakpoints upstream of exon 11 retain the PHD domain I structurally intact for the reciprocal fusion protein, while breakpoint within exon 11 or downstream of it seem to result in a different folding of the PHD domain I, leading to an impairment for CYP33 binding and the homo-dimerization capacity of the PHD domain I [28, 29].

**Fig. 5:**
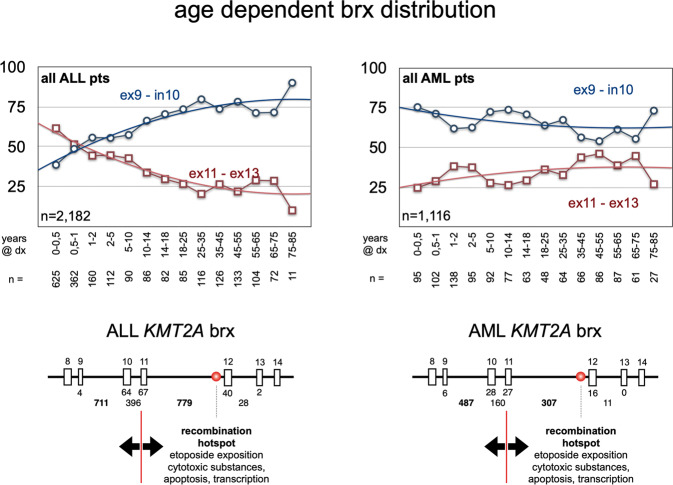
Breakpoint distribution A/B vs. C in the disease subgroups ALL and AML. The age and breakpoint distribution within the *KMT2A* gene. As indicated in Suppl. Table 3, the breakpoints in *KMT2A* distributed differently in infant, pediatric and adult patients. Here, patients were categorized by disease subtype (ALL or AML) and age at diagnoses in years (indicated under the plots). The amount of breakpoints in the regions *KMT2A* ex9-in10 (region A/B; blue lines) was compared to the breakpoints in region *KMT2A* ex11-ex13 (region C; red lines). From this analysis it became clear that ALL patients below 6 months at diagnosis have much more breakpoints in the region C than in region A/B. After 6 months, this changes into the opposite distribution with having at the end 90% of breakpoints within region A/B and only 10% of breakpoints in region C. This is completely different in AML patients, where breakpoints start already in the first months of life at 75% within regions A/B and slowly decreasing with age. Vice versa, breakpoints in region C slightly increase with age in AML patients, starting from 25% and ending in elderly patients at much higher rates. This again demonstrates that infant ALL patients up to 6 months at diagnosis are probably different from all other patients.

We also correlated the number of breakpoints within the two regions exon 9 - intron 10 and exons 11–13 with the age of individual patients that exhibited either an ALL or AML disease phenotype (Fig. 5). In both disease subgroups breakpoint tendencies seem to change with age. In ALL patients, the infant group displays a clear preference for *KMT2A* intron 11 fusions. This preference appears to switch at about 6 months, when the majority of patients display a preference for *KMT2A* intron 9 fusions. Conversely, AML patients preferentially display a *KMT2A* intron 9 breakage which is slightly decreasing with age. These “breakpoint preferences” in the two disease subgroups and their change with age is potentially indicating that “infant ALL” (<6 months) is representing a unique group, which differs from pediatric and adult ALL. Most likely, “infant ALL” - especially cells with t(4;11)/*KMT2A::AFF1* translocations - derives from rapidly growing proB fetal liver cells (CD10^-^, CD19^+^, CD34^+^), while all other disease subgroups derive from bone marrow hematopoietic stem/precursor cells [30, 31].

Finally, we had also 61 patients diagnosed with MPAL (mixed phenotype acute leukemia). These patients distributed into 35 infants (*AFF1* (*n* = 12), *EPS15* (*n* = 2), *MLLT1* (*n* = 6), *MLLT10* (*n* = 5), *MLLT3* (*n* = 5), *USP2* (*n* = 4), no der(11) fusion allele), 16 pediatric (*AFF1* (*n* = 6), *CT45A1*, *ELL*, *MLLT3*, *RABGAP1*, *USP2* (*n* = 4), *USP8*, 11q23) and 10 adult patients (*AFDN*, *ARHGEF12*, *ELL*, *EPS15*, *MLLT1* (*n* = 2), *MLLT3* (*n* = 2), *KMT2A*-PTD (*n* = 2)), They represented 1.8% of all diagnosed patients.

### The minor BCR of *KMT2A*

A total of 47 breakpoints were found in the minor BCR of *KMT2A*. The most frequent partner genes are *USP2* (*n* = 29), *AFDN* (*n* = 9), and *USP8* (*n* = 3). Other fusions have been identified only once comprising *AFF1, ARHGAP32*, *CREBBP, ELL, MLLT3* and *MLLT10*. *USP2* cases were mainly associated with B-ALL (*n* = 21) and MPAL (*n* = 7), while one patient was diagnosed with AML. The *AFDN* involving cases were mainly associated with T-ALL (*n* = 8), while one patient was diagnosed with B-ALL. All other cases displayed an ALL (*n* = 7) or MPAL (*n* = 2) disease phenotype.

Noteworthy, in the major BCR the four most frequent partner genes *AFF1*, *MLLT3*, *MLLT1*, and *MLLT10* are responsible for 80% of the cases. By contrast, partner genes identified in the minor BCR were *USP2*, *AFDN*, and *USP8*, which account for 85% of these cases. While *USP2* and *USP8* are exclusively found in the minor BCR, the others are found in BCR1 and BCR2.

### T-ALL cases

A fraction of investigated patients was diagnosed with a T-ALL (*n* = 123). This group of patients is mainly characterized by *KMT2A* fusions with *MLLT1* (*n* = 43) and *AFDN* (*n* = 40). Other fusions were *AFF1* (*n* = 4)*, CBL* (*n* = 5), *CLIP2*, *CLTC*, *CREBBP*, *KNL1*, *MLLT3* (*n* = 7)*, MLLT6, MLLT10* (*n* = 3)*, MAML2* (*n* = 2)*, PFDN4, PRPF19* (*n* = 2), *RUNDC3B, SEPT5, SEPT11* (*n* = 2)*, STK4, TNRC18* (*n* = 4), one reciprocal *MPZL3::KMT2A* fusion and one arbitrary fusion with no identified fusion partner gene. From this T-ALL cohort nine *KMT2A::AFDN* and one *KMT2A::CREBBP* patient breakpoints were identified in the minor BCR. This is quite important because these minor BCR breakpoints include the complete PHD1–3, the BD domain as well as the complete ePHD4 domain of KMT2A into the direct KMT2A fusion protein with AFDN. The PHD1–3 and bromodomain exert an important regulatory function when binding the isoprolylisomerase CYP33. This enables the recruitment of a Polycomb repressor complex to the CXXC domain localized in the 5’-KMT2A portion, which in turn is reversing the function of KMT2A from a transcriptional activator into a transcriptional repressor. This quite interesting situation has been recently investigated in an experimental fashion, demonstrating that the swap of the PHD1–4 domain between direct and reciprocal KMT2A fusion proteins (KMT2A, AFDN) have an enormous impact on the biological functions of these different KMT2A fusion proteins [32].

### Therapy-induced leukemia

We also investigated patients with therapy-induced leukemia (TIL; *n* = 142). A total of 34 were t-ALL cases, 101 were t-AML cases, while 7 other therapy-induced leukemia patients displayed either MPAL (*n* = 2), MDS (*n* = 3), NHL, or no disease classification (NA). The dominant partner genes were *MLLT3* (*n* *=* 49), *AFF1* (*n* = 20), *ELL* (*n* = 14), and *MLLT1* (*n* = 10). The following fusion partner genes were found in t-ALL cases: *ACTN4*, *AFDN*, *AFF1* (*n* = 20), *CEP164*, *CREBBP*, *EPS15*, *FOXO3* (*n* = 2), *MAML2* (*n* = 3), *MLLT1*, *MLLT10*, *PRCC1*, 21q22; in t-AML the following fusion partners were diagnosed: *ACTN4*, *AFDN* (*n* = 3), *AKAP13*, *ARHGEF12* (*n* = 2), *CBL*, *CEP170B* (*n* = 2), *CREBBP*, *ELL* (*n* = 13), *EPS15*, *GMPS*, *KNL1*, *LAMC3*, *ME2*, *MLLT1* (*n* = 9), *MLLT10* (*n* = 3), *MLLT3* (*n* = 47), *PDS5A*, *KMT2A*-PTD (*n* = 5), *SEPTIN11*, *SEPTIN9* (*n* = 2), *SNX9*, *TCF12*, *TET1* and (inv)11p12. As expected, TILs were mostly diagnosed in pediatric (*n* = 52) and adult patients *(n* = 88), while only one patient was found in the infant cohort. One patient had no age classification at diagnosis (NA). The gender distribution was 73 females and 69 male patients.

### Spliced fusions

Spliced fusions are generated by recombination events where the 5’-portion of the *KMT2A* gene is located upstream of an intact fusion partner gene. Since there is no transcription termination downstream of the translocated 5’-*KMT2A* gene fragment, transcripts starting at the *KMT2A* promoter are transcribing into the downstream located fusion partner gene. In this case, the most 3’-exon of the 5’-*KMT2A* is usually splicing to exon 2 the this fusion partner gene, as this exon is the first to exhibit a bona fide splice acceptor site. Spliced fusions are rare events except for *MLLT1 gene fusions* (*n* = 460), where about 50% of the breakpoints (*n* = 241) localize upstream of *MLLT1* exon 1. In these cases, only *KMT2A::MLLT1* fusion transcripts but no reciprocal one are expressed [33]. A similar scenario was found in other cases where a truncated *KMT2A* was recombined upstream of *EPS15* (12 out of 51 cases), *ELL* (10 out of 119 cases), *PRPF19* (3 out of 3 cases), *AFDN* (2 out of 138 cases), *MLLT3* (2 out of 612 cases), *AFF1* (1 out of 1262 cases), *DCPS* (1 out of 1), *MYO1F* (1 out of 5 cases), *SEPTIN5* (1 out of 1), and *CT45A2* (1 out of 1). A total of 275 cases using this mechanism were identified.

### The *KMT2A* recombinome

Based on the results obtained in the present and previous studies [13–17], a total of 79 direct TPGs and their specific breakpoint regions have been identified, all of which generate an in-frame KMT2A fusion protein (Table 1, #1–79). Another 15 TPGs were fused out-of-frame to the 5’-end of the *KMT2A* gene (Table 1, #80–94, Table 2B). For 16 patients no direct *KMT2A* fusion gene could be identified, of which 9 cases had reciprocal *KMT2A* fusion genes (*DLAT, DSCAML1, KMD2A, MACF1, RELA, RNF25, RNF115, SORL1 and ZNF57*) and 7 patients were without a reciprocal TPG; (Table 1: #95, Table 2F). Eighteen *KMT2A*-r patients showed a translocation with a chromosomal locus where to date no gene has been identified (Table 1, #96). In two cases, a reciprocal *RORA::KMT2A* (20p13) and a *DLG2::KMT2A* fusion gene (10p11.1) was identified (Table 1, #97, Table 2C), while the 5’-*KMT2A* portion was deleted. (Table 2D). In one case, the insertion of *KMT2A* material was found between *ETV6* and *RUNX1*. (Table 1, #98, Table 2E). Therefore, these 37 *KMT2A* rearrangements (Table 1, #95–98) probably represent a subclass of *KMT2A* abnormalities for which other genetic abnormalities may account for the transformed phenotype of the leukemia cells [34, 35].

**Table 2:** Overview about the MLL recombinome 2023.

#	Cytogenetic abnormality	Breakpoint	TPG	Reference	Leukemia-Type
**A**.	**in-frame KMT2A-fusion**				
1	t(1;11)(p32.3;q23.3)	1p32.3	EPS15	Bernard et al. (1994)	ALL, MPAL, AML, CML, MDS
2	t(1;11)(q21.3;q23.3)	1q21.3	MLLT11	Tse et al. (1995)	AML,t-AML, ALL,t-ALL, MPAL
3	ins(1;11)(q42.12;q23.3)	1q42.12	ENAH	Lavallée et al. (2015)	AML
4	t(1;11)(q43;q23.3)	1q43	ACTN2	KMT2A recombinome 2023	ALL
5	t(2;11)(p23.3;q23.3)	2q23.3	ASXL2	Haferlach et al. (2016)	t-AML
6	ins(2;11)(q11.2;q23.3)	2q11.2	AFF3	von Bergh et al. (2002)	ALL, T-ALL
7	t(2;11)(q33.2;q23.3)	2q33.2	ABI2	Coenen et al. (2012)	AML
8	t(2;11)(q37.3;q23.3)	2q37.3	SEPTIN2	Cerveira et al. (2006)	t-AML, AML, t-MDS
9	t(2;11)(q37.1;q23.3)	2q37.1	GIGYF2	Meyer et al. (2018)	ALL
10	t(3;11)(p21.31;q23.3)	3p21.31	NCKIPSD	Sano et al. (2000)	t-AML
11	t(3;11)(p21.1;q23.3)	3p21.1	DCP1A	Meyer et al. (2008)	ALL, AML
12	t(3;11)(q13.13;q23.3)	3q13.13	CIP2A	Coenen et al. (2011)	AML
13	t(3;11)(q25.31;q23.3)	3q25.31	GMPS	Pegram et al. (2000)	t-AML, t-MDS
14	t(3;11)(q28;q23.3)	3q28	LPP	Daheron et al. (2001)	t-AML
15	t(4;11)(p14;q23.3)	4p14	PDS5A	Meyer et al. (2011)	t-AML, AML
16	t(4;11)(p11;q23.3)	4p11	FRYL	Hayette et al. (2006)	t-ALL, t-AML, t-MDS
17	t(4;11)(q21.1;q23.3)	4q21.1	USO1	Wen et al. (2020)	t-AML
18	t(4;11)(q21.1;q23.3)	4q21.1	SEPTIN11	Kojima et al. (2004)	T-ALL, CML, t-ALL, t-AML
19	t(4;11)(q21.3-q22.1;q23.3)	4q21.3-q22.1	AFF1	Gu et al. (1992)	ALL, t-ALL, MPAL, AML
20	ins(4;11)(q22.1;q23.3)	4q22.1	FAM13A	KMT2A recombinome 2023	ALL
21	ins(4;11)(q35.1;q23.3)	4q35.1	SORBS2	Pession et al. (2006)	AML
22	t(5;11)(q12.1;q23.3)	5q12.1	KIF2A	Meyer et al. (2018)	ALL
23	ins(5;11)(q12.3;q23.3)	5q12.3	CENPK	Taki et al. (1996)	AML
24	t(5;11)(q23.2;q23.3)	5q23.2	PRRC1	Douet-Guilbert et al. (2014)	t-ALL
25	ins(5;11)(q31.1;q23.3)	5q31.1	AFF4	Taki et al. (1999)	ALL
26	t(5;11)(q31.2;q23.3)	5q31.2	MATR3	KMT2A recombinome 2023	ALL
27	t(5;11)(q31.3;q23.3)	5q31.3	ARHGAP26	Borkhardt et al. (2000)	JMML, AML
28	t(5;11)(q35.3;q23.3)	5q35.3	MAML1	Tandon et al. (2020)	ALL
29	t(6;11)(q13;q23.3)	6q13	SMAP1	Meyer et al (2005)	AML
30	t(6;11)(q15;q23.3)	6q15	CASP8AP2	Park et al (2009)	AML
31	t(6;11)(q21;q23.3)	6q21	FOXO3	Hillion et al. (1997)	t-AML, t-ALL
32	t(6;11)(q25.3;q23.3)	6q25.3	SNX9	Meyer et al. (2019)	t-AML
33	t(6;11)(q27;q23.3)	6q27	AFDN	Prasad et al. (1993)	AML, t-AML, ALL, T-ALL
34	t(7;11)(p22.1;q23.3)	7p22.1	TNRC18	Meyer et al. (2009)	T-ALL, ALL
35	t(7;11)(p11.2;q23.3)	7p11.2	SEPTIN14	Nguyen et al. (2017)	AML
36	t(7;11)(q11.23;q23.3)	7q11.23	CLIP2	Meyer et al. (2018)	ALL
37	t(7;11)(q21.12;q23.3)	7q21.12	RUNDC3B	Meyer et al. (2013)	T-ALL
38	t(7;11)(q32.1;q23.3)	7q32.1	FLNC	Haferlach et al. (2016)	t-AML
39	t(7;11)(q32.1;q23.3)	7q32.1	AHCYL2	KMT2A recombinome 2023	AML
40	t(7;11)(q36.1;q23.3)	7q36.1	GIMAP8	Berg et al. (2021)	AUL
41	ins(9;11)(p13.3;q23.3)	9p13.3	CLTA	Meyer et al. (2018)	ALL
42	t(9;11)(p21.3;q23.3)	9p21.3	MLLT3	Nakamura et al. (1993)	AML, t-AML, ALL, T-ALL, MPAL
43	t(9;11)(q33.2;q23.3)	9q33.2	DAB2IP	von Bergh et al. (2004)	AML
44	t(9;11)(33.2-q33.3;q23.3)	9q33.2-q33.3	RABGAP1	Meyer et al. (2018)	ALL
45	ins(9;11)(q34.11;q23.3)	9q34.11	FNBP1	Fuchs et al. (2001)	AML
46	t(9;11)(q34.12;q23.3)	9q34.12	LAMC3	Meyer et al. (2009)	t-AML
47	t(10;11)(p12.31;q23.3)	10p12.31	NEBL	Cóser et al. (2010)	AML
48	ins(10;11)(p12.31;q23.3)	10p12.31	MLLT10	Chaplin et al. (1995)	AML, t-AML, ALL, T-ALL, MPAL
49	t(10;11)(p12.1;q23.3)	10p12.1	ABI1	Taki et al. (1998)	AML
50	t(10;11)(q21.3;q23.3)	10q21.3	TET1	Ono et al. (2002)	AML, t-AML, ALL
51	t(10;11)(q23.2;q23.3)	10q23.2	NUTM2A	Zerkalenkova et al. (2021)	T-ALL
52	inv(11)(p15.4q23.3)	11p15.4	NRIP3	Balgobind et al. (2009)	AML
53	inv(11)(q12.1q23.3)	11q12.1	BTBD18	Alonso et al. (2012)	ALL
54	inv(11)(q12.2q23.3)	11q12.2	PRPF19	Meyer et al. (2013)	T-ALL, ALL, AML
55	t(11;11)(q13.4;q23.3)	11q13.4	ARHGEF17	Teuffel et al. (2005)	AML
56	inv(11)(q13.4q23.3)	11q13.4	C2CD3	Meyer et al. (2009)	AML
57	inv(11)(q14.2q23.3)	11q14.2	PICALM	Wechsler et al. (2003)	AML, ALL
58	inv(11)(q21q23.3)	11q21	MAML2	Meyer et al. (2006)	T-ALL,t-T-ALL, t-AML, t-MDS
59	del(11)(q23.3q23.3)	11q23.3	CBL	Fu et al. (2003)	AML, t-AML, T-ALL, ALL
60	inv(11)(q23.3q23.3)	11q23.3	USP2	Roberts et al. (2014)	MPAL, ALL, AML
61	del(11)(q23.3q23.3)	11q23.3	ARHGEF12	Kourlas et al. (2000)	AML, t-AML, t-ALL
62	del(11))(q23.3q24.2)	11q24.2	DCPS	Meyer et al (2005)	AML
63	t(11;12)(q23.3;p11.23)	12p11.23	ITPR2	Haferlach et al. (2016)	t-MDS
64	t(11;12)(q23.3;q13.2)	12q13.2	SARNP	Hashii et al. (2004)	AML
65	t(11;12)(q23.3;q14.1)	12q14.1	MON2	Gong et al. (2021)	t-AML
66	t(11;14)(q23.3;q23.3-q24.1)	14q23.3-q24.1	GPHN	Kuwada et al. (2001)	AML, t-AML
67	t(11;14)(q23.3;q32.33)	14q32.33	CEP170B	Meyer et al (2006)	t-AML
68	t(11;15)(q23.3;q15.1)	15q15.1	KNL1	Hayette et al. (2000)	AML, t-MDS, ALL, T-ALL
69	t(11;15)(q23.3;q15.1)	15q15.1	ZFYVE19	Chinwalla et al. (2003)	AML
70	t(11;15)(q23.3;q21.2)	15q21.2	USP8	Meyer et al. (2019)	ALL, MPAL
71	t(11;15)(q23.3;q21.3)	15q21.3	TCF12	Meyer et al. (2018)	t-AML
72	t(11;15)(q23.3;q25.3)	15q25.3	AKAP13	Meyer et al. (2013)	t-AML
73	t(11;16)(q23.3;p13.3)	16p13.3	CREBBP	Taki et al. (1997)	t-MDS, MDS, t-AML, AML, t-ALL, t-CML, t-T-NHL, t-T-ALL
74	t(11;16)(q23.3;p13.11)	16p13.11	MYH11	Meyer et al. (2013)	AML
75	t(11;16)(q23.3;q22.1)	16q22.1	EDC4	Becker et al. (2019)	AML
76	t(11;16)(q23.3;q24.1)	16q24.1	USP10	Zerkalenkova et al. (2018)	AML
77	t(11;17)(q23.3;p13.1)	17p13.1	GAS7	Megonigal et al. (2000)	t-AML
78	t(11;17)(q23.3;p11.2)	17p11.2	TOP3A	Herbaux et al. (2012)	AML
79	ins(11;17)(q23.3;q12)	17q12	ACACA	Meyer et al (2005)	AML
80	t(11;17)(q23.3;q12)	17q12	MLLT6	Prasad et al. (1994)	AML, ALL, T-ALL
81	t(11;17)(q23.3;q12)	17q12	LASP1	Strehl et al. (2003)	AML
82	t(11;17)(q23.3;q23.1)	17q23.1	CLTC	Meyer et al. (2018)	AML, ALL, T-ALL
83	t(11;17)(q23.3;q25.3)	17q25.3	SEPTIN9	Osaka et al. (1999)	t-AML, AML, MDS, ALL
84	t(11;18)(q23.3;q21.2)	18q21.2	ME2	Szotkowski et al. (2015)	t-AML
85	t(11;19)(q23.3;p13.3)	19p13.3	SH3GL1	So et al. (1997)	AML
86	t(11;19)(q23.3;p13.3)	19p13.3	RANBP3	KMT2A recombinome 2023	ALL
87	t(11;19)(q23.3;p13.3)	19p13.3	MLLT1	Tkachuk et al. (1992)	ALL, T-ALL, AML, MPAL, t-AL
88	t(11;19)(q23.3;p13.3)	19p13.3	ACER1	Lo Nigro et al. (2002)	ALL
89	ins(11;19)(q23.3;p13.3)	19p13.3	VAV1	Meyer et al. (2009)	AML
90	t(11;19)(q23.3;p13.2)	19p13.2	MYO1F	Lo Nigro et al. (2002)	AML
91	t(11;19)(q23.3;p13.11)	19p13.11	ELL	Thirman et al. (1994)	ALL, MPAL, AML, t-AML
92	t(11;19)(q23.3;q13.2)	19q13.2	ACTN4	Burmeister et al. (2009)	t-ALL, t-AML
93	t(11;20)(q23.3;q11.21)	20q11.21	MAPRE1	Fu et al. (2005)	ALL
94	t(11;20)(q23.3;q13.12)	20q13.12	STK4	KMT2A recombinome 2023	T-ALL
95	t(11;20)(q23.3;q13.13)	20q13.13	BCAS4	KMT2A recombinome 2023	ALL
96	t(11;20)(q23.3;q13.2)	20q13.2	PFDN4	Meyer et al. (2018)	T-ALL
97	t(11;21)(q23.3;q22.11)	21q22.11	ITSN1	KMT2A recombinome 2023	AML
98	t(11;22)(q23.3;q11.21)	22q11.21	SEPTIN5	Megonigal et al. (1998)	AML, T-ALL, ALL
99	t(11;22)(q23.3;q13.1–13.2)	22q13.1–13.2	MRTFA	Meyer et al. (2018)	ALL
100	t(11;22)(q23.3;q13.2)	22q13.2	EP300	Ida et al. (1997)	t-AML, t-MDS
101	t(11;22)(q23.3;q13.2)	22q13.2	SEPTIN3	Meyer et al (2019)	t-AML
102	t(X;11)(p11.23;q23.3)	Xp11.23	TFE3	Kosasih et al (2020)	ALL
103	t(X;11)(q13.1;q23.3)	Xq13.1	FOXO4	Parry et al. (1994)	T-ALL, ALL, t-ALL,CLL, AML
104	ins(X;11)(q22.1;q23.3)	Xq22.1	BTK	Zerkalenkova et al. (2021)	AML
105	ins(X;11)(q24;q23.3)	Xq24	SEPTIN6	Borkhardt et al. (2001)	AML
106	ins(X;11)(q26.3;q23.3)	Xq26.3	CT45S2	Cerveira et al. (2010)	MPAL
107	ins(X;11)(q28;q23.3)	Xq28	FLNA	De Braekeleer et al. (2009)	AML
**B**.	**out-of-frame KMT2A-fusion**		**TPG/ rec.TPG**		
1	t(3;11)(p21.31;q23.3)	3p21.31	SACM1L/SACM1L	Mori et al (2010)	N/A
2	t(3;11)(q21.3;q23.3)	3q21.3	EEFSEC/?	Meyer et al (2005)	ALL
3	t(6;11)(p22.3;q23.3)	6p22.3	NUP153/RIPQR2	Meyer et al. (2018)	ALL
4	t(8;10;11)(q22.3q26.3;q23.3)	10q26.3	MGMT/RRM2B	KMT2A recombinome 2023	ALL
5	complex	9q21.33	DAPK1/KMT2A	KMT2A recombinome 2023	AML
6	inv(11)(p15.5q23.3)	11p15.5	AP2A2/AP2A2	Meyer et al. (2013)	AML
7	complex	11q14.3	NOX4/?	Meyer et al. (2018)	AML
8	inv(11)(q23.3q23.3)	11q23.3	BUD13/del	Meyer et al. (2013)	AML
9	t(11;15)(q23.3q;q21)	11q23.3	LOC100131626/TCF12	Meyer et al. (2011)	MDS
10	del(11)(q23.3q23.3)	11q23.3	CEP164/del	Meyer et al. (2013)	t-ALL
11	del(11)(q23.3q23.3)	11q23.3	DDX6/del	KMT2A recombinome 2023	ALL
12	del(11)(q23.3q23.3)	11q23.3	BCL9L/del	Meyer et al (2006)	ALL
13	del(11)(q23.3q24.3)	11q24.3	ARHGAP32/del	KMT2A recombinome 2023	t-ALL
14	del(11)(q23.3q24.3)	11q25	OPCML/del	KMT2A recombinome 2023	ALL
15	t(2;11;19)(p23.3;q23.3;p13.3)	19p13.3	RANBP3-DT/PPM1G	Meyer et al. (2009)	AML
16	t(11;19)(q23.3;p13.3)	19p13.3	MIDN/del	KMT2A recombinome 2023	ALL
**C**.	**No partner gene fused to 5’-KMT2A**		**TPG/ rec.TPG**		
1	t(1;11)(p13.1;q23.3)	1p13.1	1p13.1/1p13.1	Meyer et al. (2013)	t-PMF
2	t(4;11)(q31.3;q23.3)	4q31.3	4q31.3/4q31.3	KMT2A recombinome 2023	ALL
3	t(6;11)(q27;q23.3)	6q27	6q27/?	Meyer et al. (2018)	AML
4	t(7;11)(q22.3;q23.3)	7q22.3	7q22.3/7q22.3	KMT2A recombinome 2023	ALL
5	t(9;11)(p13.3;q23.3)	9p13.3	9p13.3/FAM219A	Meyer et al. (2013)	t-ALL
6	ins(10;11)(p12.31;q14.1q23.3)	10p11.1	10p11.1/DLG2	KMT2A recombinome 2023	ALL
7	del(11)(q23.3q23.3)	11q23.3	11q23.3/del	Meyer et al. (2013)	ALL
8	inv(11)(q23.3q23.3)	11q23.3	11q23.3/?	Meyer et al. (2013)	AML
9	inv(11)(q23.3q23.3)	11q23.3	11q23.3/?	Meyer et al. (2018)	ALL
10	del(11)(q23.3q23.3)	11q23.3	11q23.3/del	Meyer et al. (2013)	ALL
11	del(11)(q23.3q23.3)	11q23.3	11q23.3/del	Meyer et al. (2013)	AML
12	del(11)(q23.3q23.3)	11q23.3	11q23.3/del	KMT2A recombinome 2023	MPAL
13	del(11)(q23.3q23.3)	11q23.3	11q23.3/del	KMT2A recombinome 2023	t-AML
14	del(11)(q23.3q23.3)	11q23.3	11q23.3/del	KMT2A recombinome 2023	T-ALL
15	inv(11)(q23.3q24.3)	11q24.3	11q24.3/11q24.3	Meyer et al. (2013)	AML
16	inv(11)(p12q23.3)	11p12	11p12/11p12	KMT2A recombinome 2023	t-AML
17	complex case	20p13	20p13/RORA	KMT2A recombinome 2023	ALL
18	t(11;21)(q23.3;q11.21)	21q22	21q22/21q22	Meyer et al. (2013)	t-ALL
**D**.	**5’-KMT2A deleted**		**rec. TPG**		
1	del(11)(q23.3q23.3)	11q23.3	MPZL3	KMT2A recombinome 2023	T-ALL
2	del(11)(q23.3q23.3)	11q23.3	IFT46	KMT2A recombinome 2023	AML
**E**.	**KMT2A inserted into another translocation**				
1	RUNX1-ETV6 with KMT2A insertion			KMT2A recombinome 2023	LBL
**F**.	**complex cases with reciprocal fusion genes**				
1	16 cases with no direct KMT2A-TPG identified / only reciprocal fusion	KMT2A recombinome 2023	ALL, AML, MPAL		

List of yet characterized direct 107 TPGs (**A**), their chromosome localization, gene name, the appropriate literature reference and observed disease phenotype(s). Genes marked with “KMT2A recombinome 2023” are new. **B**. 16 *KMT2A* rearrangements were identified with an out-of-frame fusion; **C**. 18 patients had no partner gene fused to 5’-*KMT2A*. Those fusions occurred with intergenic chromosomal regions, however, in three cases a reciprocal fusion gene was identified. **D**. 2 patients had a 5’-*KMT2A* deletion. Both reciprocal gene fusions were out-of-frame. **E**. One patient had an *ETV6-RUNX1* fusion with a *KMT2A* insertion at the breakpoint; F. For 16 patients the der(11) could not be identified, however, a reciprocal fusion allele was diagnosed.

In our cohort of 3401 patients, a total of 426 patients displayed complex rearrangements involving *KMT2A*. Within this group of patients, a total of 40 reciprocal *KMT2A* fusions represent in-frame fusions, while 386 fusions were either non-functional or out-of-frame gene fusions at the genomic DNA level (167 chromosome loci / 219 partner genes, see also Suppl. Table S4).

These 40 in-frame and 219 out-of-frame reciprocal TPGs (bearing the *KMT2A* C-terminus) were identified in complex rearrangements with already known direct fusion partner genes (*ACNT2, AFDN, AFF1, AFF3, ELL, MLLT1, MLLT10, MLLT3, MLLT6 and SEPTIN6* for the 40 in-frame fusions; *ABI1, AFDN, AFF1, AFF3, DAPK1, ELL, EPS15, FLNA, IFT46, LOC100131626, MGMT, MLLT1, MLLT3, MLLT10, MLLT11, MYO1F, PICALM, RNABP3-DT, RUNX1, SEPTIN6, SEPTIN9, TRNC18, USP2, USP8 and VAV1*). It is noteworthy that the majority of these reciprocal *KMT2A* out-of-frame fusions are per se able to express only the 3’-*KMT2A* portion, named KMT2A* protein, due to a gene internal promotor located upstream of *KMT2A* exon 12 [26].

### Novel translocation partner genes

Apart from the many new *KMT2A* fusion genes that have already been discovered at the DCAL and published in the last decade (see Table 2; 38 in-frame-fusions, 9 out-of-frame fusions, 6 chromosome loci), we present additional eight novel in-frame fused TPGs and four out-of frame fused TPGs (marked as “KMT2A recombinome 2023”). The in-frame *KMT2A* fusion partners are: *ACTN2* (1q43; actinin alpha 2; 21 exons; 894 aa), *FAM13A* (4q22.1; Family with sequence similarity 13 member A; 24 exons; 1023 aa), *MATR3* (5q35.3; Matrin3; 17 exons; 509 aa), *SNX9* (6q25.3; sorting nexin 6; 13 exon; 406 aa), *RANBP3* (19p13.3; RAN binding protein 3; 17 exons; 567 aa), STK4 (20q13.12; serine/threonine kinase 4; 12 exons; 462 aa), *BCAS4* (20q13.13; breast carcinoma amplified sequence 4; 5 exons; 173 aa) and *ITSN1* (22q11.21; intersectin 1; 40 exons; 1721 aa); the out-of-frame fusion genes are: *DDX6* (11q23.3; DEAD-box helicase 6 (inversion); 14 exons; 483 aa), *OPCML* (11q25; opioid binding protein/cell adhesion molecule like (inversion); 7 exons; 345 aa), *MGMT* (10q26.3; O-6-methylguanine-DNA methyltransferase; 5 exons; 238 aa) and *ARHGAP32* (11q24; Rho GTPase activating protein 32 (inversion); 22 exons; 2087 aa).

Table 2 also lists *KMT2A* fusion genes that have been identified by others since the last recombinome paper in 2018. These were *USO1* (4q21.1; USO1 vesicle transport factor; 26 exons; 973 aa), *MAML1* (5q35.3; mastermind like transcriptional coactivator 1; 5 exons, 1016 aa), *AHCYL2* (7q32.3; adenosylhomocysteinase like 2; 17 exon; 611 aa), *GIMAP8* (7q36.1; GTPase, IMAP family member 8; 5 exons; 665 aa), *NUTM2A* (10q23.2; NUT family member 2A; 7 exons; 878 aa), *MON2* (12q14.1; MON2 homolog, regulator of endosome-to-Golgi trafficking; 34 exons; 1711 aa), *EDC4* (16q22.1; enhancer of mRNA decapping 4; 29 exons; 1401 aa), *USP10* (16q24.1; ubiquitin specific peptidase 10; 14 exons; 798 aa), *TFE3* (Xp11.23; transcription factor binding to IGHM enhancer 3; 10 exons; 575 aa) and *BTK* (Xq22.1; Bruton tyrosine kinase); 19 exons; 659 aa).

### Novel in-frame fusions to KMT2A

*ACTN2* (Actinin Alpha 2) is a protein coding gene. Diseases associated with *ACTN2* include cardiomyopathy with or without left ventricular noncompaction and myopathy. Congenital ACNT2 mutations are associated with structured cores and Z-line abnormalities [36]. ACTN2 encodes a muscle-specific, alpha actinin isoform that is expressed in both skeletal and cardiac muscles. Alpha actinin is an actin-binding protein with multiple roles in different cell types. In nonmuscle cells, the cytoskeletal isoform is found along microfilament bundles and adherens-type junctions, where it is involved in binding actin to the membrane. In contrast, skeletal, cardiac, and smooth muscle isoforms are localized to the Z-disc and analogous dense bodies, where they help anchor the myofibrillar actin filaments. This gene encodes a muscle-specific, alpha actinin isoform that is expressed in both skeletal and cardiac muscles. ACNT2 forms antiparallel homodimers or heterodimers with ACTN3 and interacts with ADAM12, MYOZ1, MYOZ2 and MYOZ3.

*FAM13A* (Family With Sequence Similarity 13 Member A) is a protein-coding gene. Diseases associated with *FAM13A* include polycystic kidney disease 2 with or without polycystic liver disease and interstitial lung disease 2. *FAM13A* is also implicated in chronic obstructive pulmonary disease COPD). FAM13A is predicted to be involved in the regulation of small GTPase-mediated signal transduction and to be located in the cytosol. Of interest, *FAM13A* overlaps at the C-terminal portion with a convergently expressed *FAM13A-AS* lncRNA gene. Downregulation of this particular lncRNA was associated with overexpression of miR-205–3p and downregulation of DDI2 in cervical cancers. Overexpression of *FAM13A-AS* reversed this effect and caused tumor growth impairment (growth, migration, invasion) and the induction of apoptosis [37].

*MATR3* (Matrin 3) is a protein-coding gene. Diseases associated with MATR3 include amyotrophic lateral sclerosis 21 and distal myopathy with vocal cord weakness. This gene encodes a nuclear matrix protein, which is proposed to stabilize certain messenger RNA species. Matrin 3 plays a role in transcription or may interact with other nuclear matrix proteins to form the internal fibrogranular network. In association with the SFPQ-NONO heteromeric MATR3 may play a role in the nuclear retention of defective RNAs, and is involved in the regulation of DNA virus-mediated innate immune response. It is also part of a complex that serves as a platform for IRF3 phosphorylation and subsequent innate immune response activation. Matrin 3 binds to N6-methyladenosine (m6A)-containing mRNAs, e.g. by binding to m6A-containing MYC mRNAs which may inflict with MYC protein synthesis. Among several tumors, overexpression of *MATR3* has been associated with hepatocellular carcinoma (HCC) and non-small cell lung cancer (NSCLC) stageI/II development and has tumor-suppressive activity in basal-like breast cancer [38–40]. Quite important, Matrin 3 has been described as essential for the stabilization of chromatin architecture and the regulation of differentiation processes [41].

*SNX9* (Sorting Nexin 9) is a protein-coding gene. Diseases associated with SNX9 include Wiskott-Aldrich syndrome and trichothiodystrophy 3. This gene encodes a member of the sorting nexin family. Members of this family contain a phosphoinositide binding domain, and are involved in intracellular trafficking. The encoded protein does not contain a coiled-coil region, like some family members, but instead a SRC homology domain near its N-terminus. The protein has been reported to have a variety of interaction partners, including of adaptor protein 2, dynamin, tyrosine kinase non-receptor 2, Wiskott-Aldrich syndrome-like, and ARP3 actin-related protein 3. SNX9 is implicated in several stages of intracellular trafficking, including endocytosis, macropinocytosis, and F-actin nucleation. SNX9 has been described to be important for metastasis by regulating specific surface protein patterns and RhoGTPases [42–46].

*RANBP3* (RAN Binding Protein 3) is a protein-coding gene. Among its related pathways are Degradation of ß-catenin and cytoskeletal signaling. This gene encodes a protein with a RanBD1 domain, is found in both the nucleus and cytoplasm and acts as a cofactor for XPO1/CRM1-mediated nuclear export. It is a negative regulator of TGF-beta signaling through interaction with the R-SMAD proteins, SMAD2 and SMAD3, and mediating their nuclear export. RANBP3 regulates melanoma cell proliferation and ß-Catenin import in colorectal cancer [47, 48].

*STK4* (Serine/Threonine Kinase 4, also known as *MST1*) is a protein-coding gene. Diseases associated with loss-of STK4 include T-cell immunodeficiency, recurrent infections, autoimmunity and cancer progression. The protein encoded by this gene is a cytoplasmic kinase that is structurally similar to the yeast Ste20p kinase, which acts upstream of the stress-induced mitogen-activated protein kinase cascade. STK4 has been described to regulate the Hippo pathway [49]. STK4 itself undergoes autophosphorylation and can phosphorylate myelin basic protein. A caspase-cleaved fragment of the encoded protein has also been shown to be capable of phosphorylating histone H2B. The particular phosphorylation catalyzed by this protein has been correlated with apoptosis, and it is possible that this protein induces the chromatin condensation observed in this process. Phosphorylation of YAP1 by LATS2 inhibits its translocation into the nucleus to regulate cellular genes important for proliferation, cell death, and cell migration. STK4 also phosphorylates FOXO3 upon oxidative stress, which results in its nuclear translocation and cell death initiation. Similarly, it phosphorylates also SIRT1 and inhibits SIRT1-mediated TP53 deacetylation, thereby promoting TP53-dependent transcription and apoptosis upon DNA damage. In addition, STK4 acts as an inhibitor of AKT1. Downregulation of STK4 promotes colon cancer invasion/migration [50].

*BCAS4* (Breast Carcinoma Amplified Sequence 4) is a protein coding gene. Diseases associated with BCAS4 include breast cancer. BCAS4 is either amplified, overexpressed or fused with the last two exons of BCAS3 to BCAS4 in breast cancer [51]. Overexpression of BCAS4 was also detected in endometrial cancer [52].

*ITSN1* (Intersectin 1) is a protein-coding gene. Diseases associated with ITSN1 include autosomal dominant non-syndromic intellectual disability and esophageal atresia. The protein encoded by this gene is a cytoplasmic membrane-associated protein that indirectly coordinates endocytic membrane traffic with the actin assembly machinery. In addition, ITSN1 may regulate the formation of clathrin-coated vesicles and could be involved in synaptic vesicle recycling. This protein has been shown to interact with dynamin, CDC42, SNAP23, SNAP25, SPIN90, EPS15, EPN1, EPN2, and STN2. ITSNq is PI3KC2ß-dependent and has been linked to tumorigenesis of neuroblastoma and malignant glioma [53–55].

### Novel out-of-frame fusions to KMT2A

*DDX6* (DEAD-Box Helicase 6) is a protein-coding gene. Diseases associated with DDX6 include intellectual developmental disorder with impaired language and dysmorphic facies and non-specific syndromic intellectual disability. This gene encodes a member of the DEAD box protein family. The protein is an RNA helicase found in P-bodies and stress granules, and functions in translation suppression and mRNA degradation [56]. It is required for microRNA-induced gene silencing. DDX6 is implemented in the regulation of MYC expression in gastric cancer [57]. DDX6 has also been also linked to the transfer of P-TEFb from the 7SK snRNP to the AF4 super elongation complex (SEC) [58].

*OPCML* (Opioid Binding Protein/Cell Adhesion Molecule Like) is a Protein Coding gene. Diseases associated with OPCML include ovarian cancer and hypogonadotropic hypogonadism 14 with or without anosmia. This protein is localized in the plasma membrane. The opioid binding-cell adhesion molecule encoded by the rat gene binds opioid alkaloids in the presence of acidic lipids, exhibits selectivity for mu ligands and acts as a GPI-anchored protein. Since the protein is highly conserved in species during evolution, it may have a fundamental role in mammalian systems. Differential expression or DNA methylation of OPCML has been linked to several types of cancers [59–61].

*MGMT* (O-6-Methylguanine-DNA Methyltransferase) is a protein-coding gene. Diseases associated with MGMT include oligodendroglioma and gliosarcoma. MGMT is a DNA repair protein that is involved in cellular defense against mutagenesis and toxicity from alkylating agents. The protein catalyzes the transfer of methyl groups from O(6)-alkylguanine and other methylated moieties of the DNA to its own molecule, which repairs the toxic lesions. Methylation of the *MGMT* promoter or inactivating mutations have been associated with several cancer types, including colorectal cancer, lung cancer, prostate cancer, lymphoma, glioblastoma, and astrocytoma [62–64].

*ARHGAP32* (Rho GTPase Activating Protein 32) is a protein-coding gene. GTPase-activating protein (GAP) is promoting GTP hydrolysis on RHOA, CDC42 and RAC1 small GTPases. The encoded protein may be involved in the differentiation of neuronal cells during the formation of neurite extensions. It is also involved in N-methyl-D-aspartate (NMDA) receptor activity-dependent actin reorganization in dendritic spines.

In summary, the complete “*KMT2A* recombinome 2023” is comprised by 107 in-frame fusion partner genes, 16 out-of-frame gene fusions, 18 patients with fusions to chromosomal loci, 2 patients with a *5´-KMT2A* deletion but with the presence of a reciprocal fusion allele, one patient with an *KMT2A* insertion between *ETV6* and *RUNX1*, and finally, 16 patients where a *5´-KMT2A* fusion could not be identified, but with the presence of reciprocal fusion allele (Table 2).

## Discussion

Herein, we present an updated ‘*KMT2A* recombinome 2023’ associated mainly with acute leukemia, ALL and AML. Our analyses of 3401 samples were performed by using only small amounts of genomic DNA isolated from bone marrow or peripheral blood collected at diagnosis. Of these patients, 2702 were analyzed by our well-established PCR methods [14], while 696 were analyzed by state-of-the-art targeted next-generation sequencing (NGS) of the *KMT2A* [15, 16].

The applied techniques allowed to identify direct and reciprocal *KMT2A* fusions, *KMT2A* gene-internal duplications, chromosome 11 inversions, chromosomal 11q deletions and the insertion of chromosome 11 material into other chromosomes, or vice versa, the insertion of chromatin material of other chromosomes into the BCR of the *KMT2A* gene. The different LD-PCR technologies (inverse and multiplex PCR) that have been used in the past had a discovery rate of about 95%, while the *KMT2A*-targeting NGS method has nearly a 100% discovery rate. This is in contrast to diagnostic techniques based on RNA technologies, which do neither provide patient-specific chromosomal fusion sequences that may be used for MRD studies, nor allow paired-end mRNA analysis discovery rates greater than 90% due to variability in gene transcription and bioinformatic problems. However, RNA-Seq methods provide insights into alternative splice events, which could be quite important e.g. in case of “out-of-frame” fusions (see our 389 reciprocal cases), where a genomic analysis can not provide any functional information.

Our own analyses (Table 1) and data present from the literature, we can provide an updated status about the *KMT2A* recombinome (Table 2), which is currently comprised of 107 direct in-frame *KMT2A* fusions (Table 2A), 16 direct out-of-frame *KMT2A* fusions (Table 2B), 18 *KMT2A*-r patients with a translocation with a chromosomal locus where no gene is present (Table 2C), two patients with a deletion of the 5’-*KMT2A*, but with reciprocal fusion genes (Table 2D), one *RUNX1::ETV6* patient with an *KMT2A* insertion (Table 2E), and finally, 16 cases in which no direct *KMT2A* fusion but only the reciprocal *KMT2A* fusion could be detected (Table 2F).

Moreover, we successfully extended the current knowledge by analyzing more cases with complex *KMT2A* rearrangements (*n* = 426). During these analyses a large collection of reciprocal *KMT2A* fusions was identified, of which 40 were in-frame, while 386 fusions were either non-functional or out-of-frame gene fusions at the genomic DNA level (167 chromosome loci / 219 partner genes, see Suppl. Table S4). However, the majority of the reciprocal out-of-frame *KMT2A* fusions may still be transcribed and encode a 5’-truncated KMT2A protein, termed KMT2A*, due to a gene-internal promotor upstream of *KMT2A* exon 12 [26]. This shorter version of the KMT2A protein has no ability to bind Menin1, LEDGF or MYB, but still carries all enzymatic functions and necessary domains to bind known binding proteins that carry out H4K16 acetylations (by the MOF protein) or H3K4 methylation by the SET domain complex. This aberrant KMT2A* protein complex still retains the capacity to bind, read and modify chromatin. This may also explain also the findings that this particular 5’-truncated KMT2A* protein exhibited oncogenic potential in a focus formation assay [65].

Moreover, recent studies with two reciprocal fusion proteins (AFF1::KMT2A and AFDN::KMT2A) [32, 66] demonstrated their important function as “chromatin opening protein complexes”, which subsequently allowed the corresponding direct KMT2A fusion proteins to activate ~10-fold more target genes. The tremendously increased number of deregulated genes (“gain of target genes”) changed over time in an evolutionary selection process leading to the final oncogenic gene expression signature [66]. Thus, reciprocal fusion proteins are probably key elements for the onset of pre-leukemic clones that are then be selected to overt leukemic cells by the bone marrow environment. Since this process may be initiated by reciprocal fusion proteins and even maintained after their shutdown, we can assume that they are - for some TPGs - required only for the onset of the pre-leukemic state. Shutting down their gene transcription, or even deleting these reciprocal fusion alleles may even support the manifestation of an oncogenic gene expression pattern. This is also in line with two recent publications that reported a better outcome of t(4;11)/*KMT2A::AFF1* proB ALL patients when both the direct and the reciprocal fusion alleles were expressed [67, 68]. If a given transcriptome is strongly enhanced by the presence of the reciprocal fusion protein, then this also causes the expression of more druggable target proteins. Under chemotherapy this may translate in better outcome, because more druggable targets may result in a higher sensitivity of these tumor cells. Thus, leukemia cells with a missing reciprocal fusion protein and displaying therefore a more restricted transcriptome could potentially exhibit a more resistant phenotype.

**Fig. 6:**
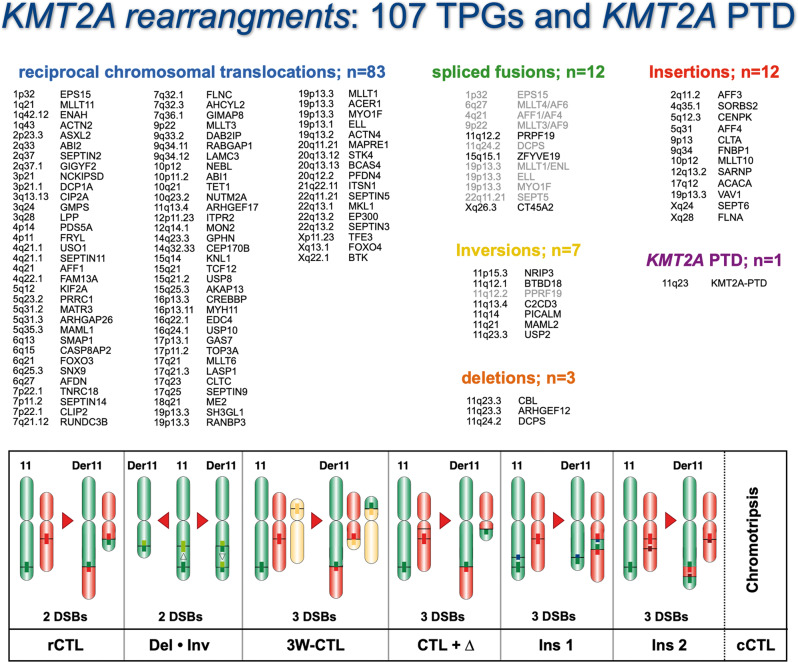
The KMT2A recombinome 2023. All known *KMT2A* gene rearrangements are subclassified either into reciprocal (balanced) chromosomal translocations (*n* = 91), spliced fusions (*n* = 3 + 9), inversion on chromosome 11 (*n* = 6 + 1), deletions on chromosome 11 *(n* = 3) and TPG chromatin fragment insertions into the *KMT2A* gene, or vice versa, *KMT2A* gene fragment insertions into the TPG’s (*n* = 12). A few of the possible gene rearrangements are depicted at the bottom where different genetic scenarios are indicated. Since we had analyzed more than 400 complex *KMT2A* rearrangements, most of these scenarios have been identified, apart from chromotripsis which is a known mechanism to generate a multitude of gene fusions in solid cancer, but not in hemato-malignant tumors.

The analysis of 3401 *KMT2A* fusion alleles over the past 20 years has led to the discovery of 48 novel TPGs (Table 2) of which 13 TPGs have not been published yet. Together with 59 TPGs described in literature, we can present today a total of 107 direct *KMT2A* fusions that have been characterized at the molecular level. We have summarized all currently known *KMT2A* TPGs in Fig. 6, according to the genetic aberration in which they have been diagnosed.

The 8 most frequent TPGs of the *KMT2A* gene are *AFF1*, *MLLT3*, *MLLT1*, *MLLT10*, *ELL, AFDN*, *EPS15* and *KMT2A*-PTDs. Their occurrence differ significantly in the cohorts of infant, pediatric and adult leukemia patients. We also observed significant differences of individual fusion genes and gender distribution (see Suppl. Table S1): *KMT2A::MLLT10* occurs more frequently in the male group of patients (p = 0.00379), while females were more affected by *KMT2A::AFF1* fusions (p = 0.00148). The most striking finding was that the breakpoint distributions differ significantly for distinct TPGs and age groups. It is well known that breakpoints in infants occur more frequently in *KMT2A* intron 11 (Suppl. Table S3). These significant preferences clearly argue for a different biology or oncological mechanism behind the fusion proteins with respect to oligomerization capacity, exerted functions or requirements for a *HOXA* signature. At least *KMT2A::AFF1* patients that have a breakpoint in (intron 9, exon 10, intron 10) of the major BCR tend to display strong *HOXA* signatures (*HOXA*^*hi*^), while breakpoints within or downstream of exon 11 display a *HOXA*^*lo*^ gene signature. Therefore, it will be of importance to analyze other *KMT2A::TPG* entities for their *HOXA* high or low signatures. Significant differences in breakpoint distribution for different TPG classes may either be linked to the requirement of *HOXA* signatures for leukemia development, or simply reflect different cells of origin, like e.g. fetal liver or definitive hematopoietic stem cells. Future analysis may help to unravel this yet underinvestigated phenomenon.

As already mentioned above, the outcome of leukemia patients has been linked to the distribution of chromosomal breakpoints within the *KMT2A* breakpoint cluster region [23]. Basically, the outcome of leukemia patients with breakpoints in *KMT2A* intron 11 was worse compared to those patients with upstream breakpoints. The cut point was exactly at the borderline between intron 10 and exon 11. A rational explanation for this observation is provided by functional studies of the PHD domain of the KMT2A protein, encoded by *KMT2A* exons 11–16. This domain is built up by PHD1, PHD2 and an enhanced PHD3 (ePHD3). This PHD domain is separated by the adjacent bromodomain (BD) and another ePHD4 domain (encode by exons 17–21). PHD3 has an important dual function, because it either binds to the CYP33/PPIE protein [69, 70] or to methylated lysine-4 residues of histone H3 [71]. Binding of PHD3 to H3K4me2/3 peptides is greatly enhanced by the adjacent bromodomain [72], but binding of the prolyl-peptidyl isomerase CYP33/PPIE confers a *cis-trans* isomerization of proline-1665. This enables the recruitment of BMI1 and associated repressor proteins (HDAC/CBX4/KDM5B) to the CXXC domain of wildtype KMT2A to repress gene transcription. Noteworthy, PHD2 and PHD3 also bind to E3-ligases (CDC34 and ASB2, respectively) which control the steady-state stability of the KMT2A protein [73, 74]. As recently shown, breakpoints within *KMT2A* intron 11 destroy the dimerization capacity of the PHD1–3 domain [28] and disables binding to the BMI1 repressor complex [75]. Thus, a breakpoint upstream of or within *KMT2A* intron 11 has functional consequences for the resulting fusion proteins, which may provide an explanation for the altered outcome of these patients [23].

An important translational aspect of this study is the establishment of patient-specific DNA fusion junction sequences that can be used for monitoring MRD by quantitative PCR techniques. Since a given *KMT2A* fusion allele is genetically stable and a mono-allelic marker for each tumor cell, a more reliable quantification and tracing of residual tumor cells becomes feasible. For each of the 3401 acute leukemia patients at least one *KMT2A* fusion allele was identified and characterized by sequencing. Several prospective studies were already initiated and first published data verified the reliability of these genomic markers for MRD monitoring [4–7]. Therefore, the use of these MRD markers will in the future contribute to a better stratification of leukemia patients which in turn will help to further improve their outcome. In particular, in infant ALL MRD monitoring has a high impact in the outcome of the patients.

For the majority of *KMT2A* TPGs identified so far, a systematic classification about their function(s) has recently been described in comprehensive detail [76]. However, further functional studies are required to elucidate the mechanisms which are causative for their leukemogenic activity. Such studies may provide the basis for developing new therapeutic strategies in the future.

## Data availablility

Patient data of our FileMaker database containing breakpoint information and data from the investigated acute leukemia patients can be made available to scientist upon request.

## Supplementary information

Supplemental data file

## References

[1] Pui CH, Gaynon PS, Boyett JM, Chessells JM, Baruchel A, Kamps W (2002). Outcome of treatment in childhood acute lymphoblastic leukaemia with rearrangements of the 11q23 chromosomal region. The Lancet.

[2] Pui CH, Chessells JM, Camitta B, Baruchel A, Biondi A, Boyett JM (2003). Clinical heterogeneity in childhood acute lymphoblastic leukemia with 11q23 rearrangements. Leukemia.

[3] Balgobind BV, Raimondi SC, Harbott J, Zimmermann M, Alonzo TA, Auvrignon A (2009). Novel prognostic subgroups in childhood 11q23/MLL-rearranged acute myeloid leukemia: results of an international retrospective study. Blood.

[4] Szczepański T, Harrison CJ, van Dongen JJ (2010). Genetic aberrations in paediatric acute leukaemias and implications for management of patients. Lancet Oncol.

[5] Burmeister T, Marschalek R, Schneider B, Meyer C, Gökbuget N, Schwartz S (2006). Monitoring minimal residual disease by quantification of genomic chromosomal breakpoint sequences in acute leukemias with MLL aberrations. Leukemia.

[6] van der Velden VH, Corral L, Valsecchi MG, Jansen MW, De Lorenzo P, Cazzaniga G (2009). Prognostic significance of minimal residual disease in infants with acute lymphoblastic leukemia treated within the Interfant-99 protocol. Leukemia.

[7] Yeoh AE, Ariffin H, Chai EL, Kwok CS, Chan YH, Ponnudurai K (2012). Minimal residual disease-guided treatment deintensification for children with acute lymphoblastic leukemia: results from the Malaysia-Singapore acute lymphoblastic leukemia 2003 study. J Clin Oncol.

[8] Johansson B, Moorman AV, Secker-Walker LM (1998). Derivative chromosomes of 11q23-translocations in hematologic malignancies. European 11q23 Workshop participants. Leukemia.

[9] Heerema NA, Sather HN, Ge J, Arthur DC, Hilden JM, Trigg ME (1999). Cytogenetic studies of infant acute lymphoblastic leukemia: poor prognosis of infants with t(4;11) - a report of the Children’s Cancer Group. Leukemia.

[10] Van der Burg M, Beverloo HB, Langerak AW, Wijsman J, van Drunen E, Slater R (1999). Rapid and sensitive detection of all types of MLL gene translocations with a single FISH probe set. Leukemia.

[11] van der Burg M, Poulsen TS, Hunger SP, Beverloo HB, Smit EM, Vang-Nielsen K (2004). Split-signal FISH for detection of chromosome aberrations in acute lymphoblastic leukemia. Leukemia.

[12] Harrison CJ, Moorman AV, Barber KE, Broadfield ZJ, Cheung KL, Harris RL (2005). Interphase molecular cytogenetic screening for chromosomal abnormalities of prognostic significance in childhood acute lymphoblastic leukaemia: a UK Cancer Cytogenetics Group Study. Br J Haematol.

[13] Burmeister T, Meyer C, Gröger D, Hofmann J, Marschalek R (2015). Evidence-based RT-PCR methods for the detection of the 8 most common MLL aberrations in acute leukemias. Leuk Res.

[14] Meyer C, Schneider B, Reichel M, Angermueller S, Strehl S, Schnittger S (2005). Diagnostic tool for the identification of MLL rearrangements including unknown partner genes. Proc Natl Acad Sci USA.

[15] Afrin S, Zhang CRC, Meyer C, Stinson CL, Pham T, Bruxner TJC (2018). Targeted Next-Generation Sequencing for Detecting MLL Gene Fusions in Leukemia. Mol Cancer Res.

[16] Meyer C, Lopes BA, Caye-Eude A, Cavé H, Arfeuille C, Cuccuini W (2019). Human MLL/KMT2A gene exhibits a second breakpoint cluster region for recurrent MLL-USP2 fusions. Leukemia.

[17] Meyer C, Schneider B, Jakob S, Strehl S, Schnittger S, Schoch C (2006). The MLL recombinome of acute leukemias. Leukemia.

[18] Meyer C, Kowarz E, Hofmann J, Renneville A, Zuna J, Trka J (2009). New insights into the MLL recombinome of acute leukemias. Leukemia.

[19] Meyer C, Hofmann J, Burmeister T, Gröger D, Park TS, Emerenciano M (2013). The MLL recombinome of acute leukemia in 2013. Leukemia.

[20] Meyer C, Burmeister T, Gröger D, Tsaur G, Fechina L, Renneville A (2018). The MLL recombinome of acute leukemias in 2017. Leukemia.

[21] Daser A, Rabbitts TH (2005). The versatile mixed lineage leukaemia gene MLL and its many associations in leukaemogenesis. Semin Cancer Biol.

[22] Krivtsov AV, Armstrong SA (2007). MLL translocations, histone modifications and leukaemia stem-cell development. Nat Rev Cancer.

[23] Emerenciano M, Meyer C, Mansur MB, Marschalek R, Pombo-de-Oliveira MS, The Brazilian Collaborative Study Group of Infant Acute Leukaemia. (2013). The distribution of MLL breakpoints correlates with outcome in infant acute leukaemia. Br J Haematol.

[24] Strissel PL, Strick R, Rowley JD, Zeleznik-Le NJ (1998). An in vivo topoisomerase II cleavage site and a DNase I hypersensitive site colocalize near exon 9 in the MLL breakpoint cluster region. Blood.

[25] Stanulla M, Wang J, Chervinsk DS, Thandla S, Aplan PD (1997). DNA cleavage within the MLL breakpoint cluster region is a specific event which occurs as part of higher-order chromatin fragmentation during the initial stages of apoptosis. Mol Cell Biol.

[26] Scharf S, Zech J, Bursen A, Schraets D, Oliver PL, Kliem S (2007). Transcription linked to recombination: a gene-internal promoter coincides with the recombination hot spot II of the human MLL gene. Oncogene.

[27] Felix CA (2001). Leukemias related to treatment with DNA topoisomerase II inhibitors. Med Pediatr Oncol.

[28] Rössler T, Marschalek R (2013). An alternative splice process renders the MLL protein either into a transcriptional activator or repressor. Pharmazie.

[29] Marschalek R (2020). The reciprocal world of MLL fusions: A personal view. Biochim Biophys Acta Gene Regul Mech.

[30] O’Byrne S, Elliott N, Rice S, Buck G, Fordham N, Garnett C (2019). Discovery of a CD10-negative B-progenitor in human fetal life identifies unique ontogeny-related developmental programs. Blood.

[31] Rice S, Jackson T, Crump NT, Fordham N, Elliott N, O’Byrne S (2021). A human fetal liver-derived infant MLL-AF4 acute lymphoblastic leukemia model reveals a distinct fetal gene expression program. Nat Commun.

[32] Kundu A, Kowarz E, Marschalek R (2021). The role of reciprocal fusions in MLL-r acute leukemia: studying the chromosomal translocation t(6;11). Oncogene.

[33] Meyer C, Burmeister T, Strehl S, Schneider B, Hubert D, Zach O (2007). Spliced MLL fusions: a novel mechanism to generate functional chimeric MLL-MLLT1 transcripts in t(11;19)(q23;p13.3) leukemia. Leukemia.

[34] Meyer C, Kowarz E, Yip SF, Wan TS, Chan TK, Dingermann T (2011). A complex MLL rearrangement identified five years after initial MDS diagnosis is causing out-of-frame fusions whithout progression to acute leukemia. Cancer Genetics.

[35] Mori T, Nishimura N, Hasegawa D, Kawasaki K, Kosaka Y, Uchide K (2010). Persistent detection of a novel MLL-SACM1L rearrangement in the absence of leukemia. Leuk Res.

[36] Ranta-Aho J, Olive M, Vandroux M, Roticiani G, Dominguez C, Johari M (2022). Mutation update for the ACTN2 gene. Hum Mutat.

[37] Qiu Z, He L, Yu F, Lv H, Zhou Y (2022). LncRNA FAM13A-AS1 Regulates Proliferation and Apoptosis of Cervical Cancer Cells by Targeting miRNA-205-3p/DDI2 Axis. J Oncol.

[38] Shen J, Shu M, Xie S, Yan J, Pan K, Chen S (2021). A Six-Gene Prognostic Risk Prediction Model In Hepatitis B Virus-Associated Hepatocellular Carcinoma. Clin Invest Med.

[39] Durślewicz J, Klimaszewska-Wiśniewska A, Jóźwicki J, Antosik P, Kozerawski K, Grzanka D (2022). Prognostic significance of MATR3 in stage I and II non-small cell lung cancer patients. J Cancer Res Clin Oncol.

[40] Yang J, Lee SJ, Kwon Y, Ma L, Kim J (2020). Tumor suppressive function of Matrin 3 in the basal-like breast cancer. Biol Res.

[41] Cha HJ, Uyan Ö, Kai Y, Liu T, Zhu Q, Tothova Z (2021). Inner nuclear protein Matrin-3 coordinates cell differentiation by stabilizing chromatin architecture. Nat Commun.

[42] Tanigawa K, Maekawa M, Kiyoi T, Nakayama J, Kitazawa R, Kitazawa S (2019). SNX9 determines the surface levels of integrin β1 in vascular endothelial cells: Implication in poor prognosis of human colorectal cancers overexpressing SNX9. J Cell Physiol.

[43] Mygind KJ, Störiko T, Freiberg ML, Samsøe-Petersen J, Schwarz J, Andersen OM (2018). Sorting nexin 9 (SNX9) regulates levels of the transmembrane ADAM9 at the cell surface. J Biol Chem.

[44] Bendris N, Schmid SL (2017). Endocytosis, Metastasis and Beyond: Multiple Facets of SNX9. Trends Cell Biol.

[45] Bendris N, Williams KC, Reis CR, Welf ES, Chen PH, Lemmers B (2016). SNX9 promotes metastasis by enhancing cancer cell invasion via differential regulation of RhoGTPases. Mol Biol Cell.

[46] Sardou-Cezar I, Lopes BA, Andrade FG, Fonseca TCC, Fernandez TS, Larghero P (2021). Therapy-related acute myeloid leukemia with KMT2A-SNX9 gene fusion associated with a hyperdiploid karyotype after hemophagocytic lymphohistiocytosis. Cancer Genet.

[47] Pathria G, Garg B, Wagner C, Garg K, Gschaider M, Jalili A (2016). RanBP3 Regulates Melanoma Cell Proliferation via Selective Control of Nuclear Export. J Invest Dermatol.

[48] Zheng CC, Liao L, Liu YP, Yang YM, He Y, Zhang GG (2022). Blockade of Nuclear β-Catenin Signaling via Direct Targeting of RanBP3 with NU2058 Induces Cell Senescence to Suppress Colorectal Tumorigenesis. Adv Sci (Weinh).

[49] Bouchard A, Witalis M, Chang J, Panneton V, Li J, Bouklouch Y (2020). Hippo Signal Transduction Mechanisms in T Cell Immunity. Immune Netw.

[50] Lin CH, Hsu TI, Chiou PY, Hsiao M, Wang WC, Chen YC (2020). Downregulation of STK4 promotes colon cancer invasion/migration through blocking β-catenin degradation. Mol Oncol.

[51] Bärlund M, Monni O, Weaver JD, Kauraniemi P, Sauter G, Heiskanen M (2002). Cloning of BCAS3 (17q23) and BCAS4 (20q13) genes that undergo amplification, overexpression, and fusion in breast cancer. Genes Chromosomes Cancer.

[52] Wang Z, Wang H, Wang Z, He S, Jiang Z, Yan C (2020). Associated analysis of PER1/TUBB2B with endometrial cancer development caused by circadian rhythm disorders. Med Oncol.

[53] Russo A, O’Bryan JP (2012). Intersectin 1 is required for neuroblastoma tumorigenesis. Oncogene.

[54] Gu F, Zhang H, Qin F, Liu X, Li W, Fu L (2015). Intersectin1-S, a multidomain adapter protein, is essential for malignant glioma proliferation. Glia.

[55] Lan C, Zhang H, Wang K, Liu X, Zhao Y, Guo Z (2022). The alternative splicing of intersectin 1 regulated by PTBP1 promotes human glioma progression. Cell Death Dis.

[56] Di Stefano B, Luo EC, Haggerty C, Aigner S, Charlton J, Brumbaugh J (2019). The RNA Helicase DDX6 Controls Cellular Plasticity by Modulating P-Body Homeostasis. Cell Stem Cell.

[57] Taniguchi K, Iwatsuki A, Sugito N, Shinohara H, Kuranaga Y, Oshikawa Y (2018). Oncogene RNA helicase DDX6 promotes the process of c-Myc expression in gastric cancer cells. Mol Carcinog.

[58] Mück F, Bracharz S, Marschalek R (2016). DDX6 transfers P-TEFb kinase to the AF4/AF4N (AFF1) super elongation complex. Am J Blood Res.

[59] Zhang T, Chen Y, Lin W, Zheng J, Liu Y, Zou J (2021). Prognostic and Immune-Infiltrate Significance of miR-222-3p and Its Target Genes in Thyroid Cancer. Front Genet.

[60] Dobre M, Salvi A, Pelisenco IA, Vasilescu F, De Petro G, Herlea V (2021). Crosstalk Between DNA Methylation and Gene Mutations in Colorectal Cancer. Front Oncol.

[61] Shao Y, Kong J, Xu H, Wu X, Cao Y, Li W (2021). OPCML Methylation and the Risk of Ovarian Cancer: A Meta and Bioinformatics Analysis. Front Cell Dev Biol.

[62] Hareedy AA, Rohim EZA, Al Sheikh SAM, Al Shereef ZAEA (2022). Immunohistochemical Expression of PD-L1 and IDH1 with Detection of MGMT Promoter Methylation in Astrocytoma. Asian Pac J Cancer Prev.

[63] Zhong S, Ren JX, Yu ZP, Peng YD, Yu CW, Deng D (2022). Predicting glioblastoma molecular subtypes and prognosis with a multimodal model integrating convolutional neural network, radiomics, and semantics. J Neurosurg.

[64] Furini HH, Fukushima KSSQ, de Nóbrega M, de Souza MF, Rodrigues MRS, de Mattos BB (2023). An MGMT Allelic Variant Can Affect Biochemical Relapse in Prostate Cancer Patients. Anticancer Res.

[65] Wächter K, Kowarz E, Marschalek R (2014). Functional characterisation of different MLL fusion proteins by using inducible Sleeping Beauty vectors. Cancer Lett.

[66] Wilhelm A, Marschalek R (2021). The role of reciprocal fusions in MLL-r acute leukemia: studying the chromosomal translocation t(4;11). Oncogene.

[67] Yang L, Ding L, Liang J, Chen J, Tang Y, Xue H (2018). Relatively favorable prognosis for MLL-rearranged childhood acute leukemia with reciprocal translocations. Pediatr Blood Cancer.

[68] Agraz-Doblas A, Bueno C, Bashford-Rogers R, Roy A, Schneider P, Bardini M (2019). Unraveling the cellular origin and clinical prognostic markers of infant B-cell acute lymphoblastic leukemia using genome-wide analysis. Haematologica.

[69] Fair K, Anderson M, Bulanova E, Mi H, Tropschug M, Diaz MO (2001). Protein interactions of the MLL PHD fingers modulate MLL target gene regulation in human cells. Mol Cell Biol.

[70] Xia ZB, Anderson M, Diaz MO, Zeleznik-Le NJ (2003). MLL repression domain interacts with histone deacetylases, the polycomb group proteins HPC2 and BMI-1, and the corepressor C-terminal-binding protein. Proc Natl Acad Sci USA.

[71] Chang PY, Hom RA, Musselman CA, Zhu L, Kuo A, Gozani O (2010). Binding of the MLL PHD3 finger to histone H3K4me3 is required for MLL-dependent gene transcription. J Mol Biol.

[72] Wang Z, Song J, Milne TA, Wang GG, Li H, Allis CD (2010). Pro isomerization in MLL1 PHD3-bromo cassette connects H3K4me readout to CyP33 and HDAC-mediated repression. Cell.

[73] Wang J, Muntean AG, Hess JL (2012). ECSASB2 mediates MLL degradation during hematopoietic differentiation. Blood.

[74] Wang J, Muntean AG, Wu L, Hess JL (2012). A subset of mixed lineage leukemia proteins has plant homeodomain (PHD)-mediated E3 ligase activity. J Biol Chem.

[75] Grow EJ, Wysocka J (2010). Flipping MLL1’s switch one proline at a time. Cell.

[76] Marschalek R (2016). Systematic classification of Mixed-Lineage Leukemia fusion partners predicts additional cancer pathways. Ann Lab Med.

