# Supplementary material for: The KMT2A recombinome of acute leukemias in 2023
Source: Leukemia. 2023 Apr 5;37(5):988–1005. doi: 10.1038/s41375-023-01877-1 (PMC10169636; doi:10.1038/s41375-023-01877-1)
Supplement: Supplementary file 1 — Supplemental data file [file 41375_2023_1877_MOESM1_ESM.docx]

**Supplemental information**

*Suppl. Table S1:* *Overview of different patient parameters for all major TPGs*

We also investigated whether the breakpoint distribution is significantly different when compared to the mean distribution (exon9/intron = 37.2%; exon10/intron10 = 19.8% and exon11-exon13 = 39.5%). Significance was tested by a 3x2 Chi Square analysis for each distribution. The p-values are given for the most frequent partners genes (*AFF1, MLLT3, MLLT1, MLLT10, ELL, KMT2A-*PTD*s, AFDN, EPS15, SEPTIN6, SEPTIN9, MLLT6* and all others (rest)). We also investigated the significance of the breakpoint distribution by subcategorizing into gender (female/male), age groups (infants, pediatric, adult or disease phenotype (ALL, AML, other). All numbers in bold represent significant differences (p-value <0,05). Noteworthy, the distribution of females and males in *AFF1* (more females) and *MLLT10* cases (more males) was significant. Therapy-induced leukemia cases were also significantly associated with elder age groups.

*Suppl. Table S2: Overview about investigated KMT2A fusion genes and their precise breakpoint distribution*

The major fusion partners genes (*AFF1, MLLT3, MLLT1, MLLT10, ELL, KMT2A*-PTDs*, AFDN, EPS15, SEPTIN6, SEPTIN9, MLLT6* and all others (rest)) analyzed at the DCAL were analyzed for their breakpoint distribution. Precise numbers of identified breakpoints between *KMT2A* intron 7 until exon 13 (major BCR), as well as breakpoints outside the major BCR and number of breakpoint within the minor BCR are listed. We also investigated the number of breakpoints in these areas of *KMT2A* when subcategorizing by gender (female/male), age groups (infants, pediatric, adult or disease phenotype (ALL, AML, other). 57 patients were not "gender-specified" and 43 patients were not "age-specified", and thus, are missing on this Table.

Suppl. Table S3: *Proving the significance of breakpoint distribution by TPG and age groups by Chi Square analyses*

We also investigated breakpoint distribution in combination with age groups (infant, pediatric and adult) to identify significances when comparing to the mean distribution (exon9/intron = 37.2%; exon10/intron10 = 19.8% and exon11-exon13 = 39.5%). Significance was again tested by a 3x2 Chi Square analysis for each distribution. The p-values are given for the most frequent partners genes (*AFF1, MLLT3, MLLT1, MLLT10, ELL, KMT2A PTD's, AFDN, EPS15, SEPTIN6, SEPTIN9, MLLT6* and all others (rest)). All numbers in bold represent significant differences (p-value <0,05) and significant changes deviating from the mean distribution are depicted by orange colored fields.

Suppl. Table S4: *Reciprocal fusion partner genes*

A. The 40 in-frame fused reciprocal fusion partner genes: B the 386 out-of-frame fused reciprocal fusion partner genes. All genes are listed in alphabetical order and some of them were recurrently fused to 3'-KMT2A.

**Supplemental Table S1**

**
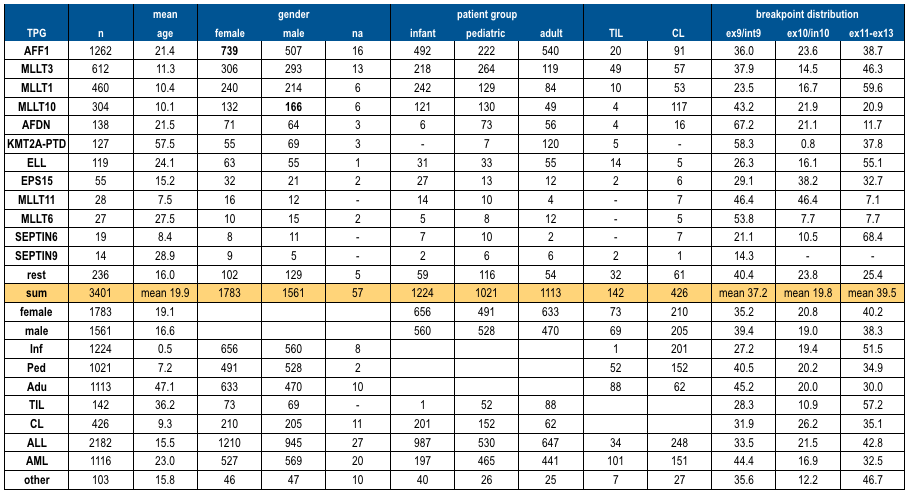
**

**Supplemental Table S2**

**
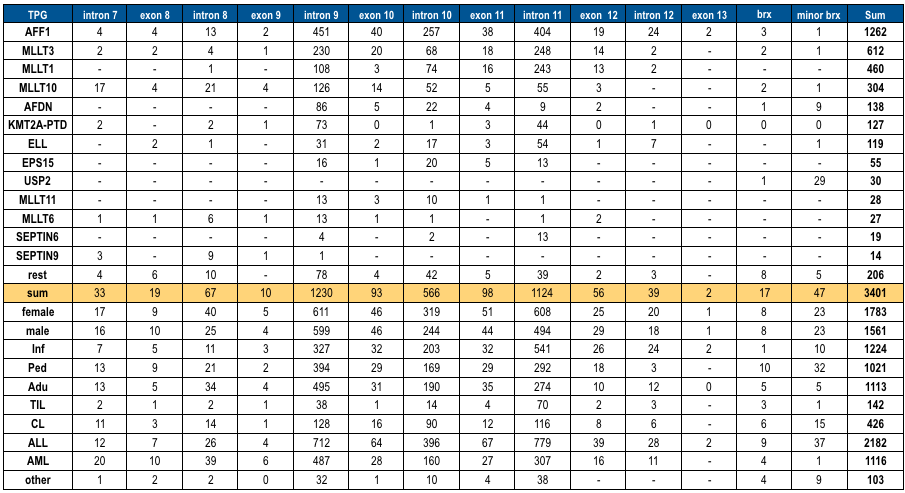
**

**Supplemental Table S3**

**
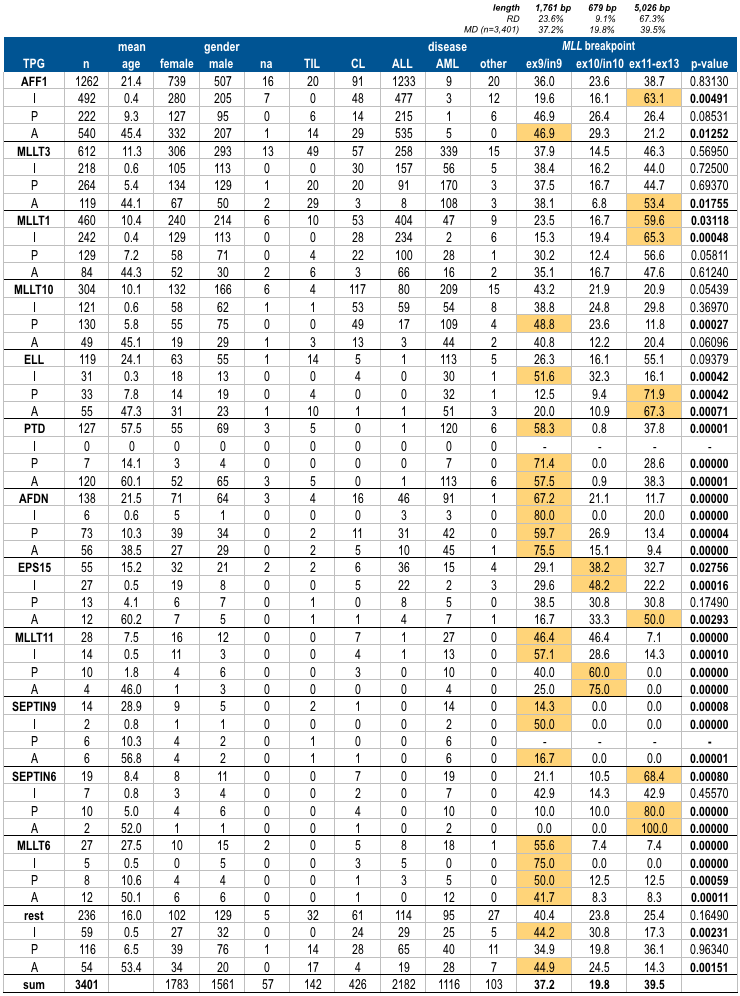
**

**Supplemental Table S4**

A. In-frame fused reciprocal TPGs (in alphabetical order)

*ACER1, ADARB2, APBB1IP, ATG16L2, ATP5MG**, C11ORF65**, CEP164 (2x), CEP164, DCP1A, DDX6, DENND4A, FGF7, GLYR1, GNA12, GUSBP16, HOATZ, ITPKB, JAK1, JAML, LRRTM4, MRPL4, MYO18A, NFKB1, NKAIN2, PAN3, PATL1, PIP4K2A, PPP1R21, PRPF6, PRPF18, RABGAP1L, RRAS2, SCAF8, SLC25A23, TECR, TRIP4, UBAP1, UVRAG, VAV1* and *WNK2.*

B. Out-of frame fused reciprocal TPG (in alphabetical order)

167 chromosome loci / 219 partner genes: *ACER1, ADSS, AFF1-AS, ANAPC10, ANKRD26, ANTXR2, ARAP1, ARCN1, ARHGAP12 (2x), ARHGEF17, ARMC3, ARPC5L, BBS12, BMP2K, BMS1P1, BTN3A1, C10ORF67, C18orf25, C2CD2L, CACNA1B, CACNB2, CBL, CBX8, CCDC171, CCDC33, CD3G, CDK14, CEACAM4, CELF1, CEP164, CEP295, CMAHP, CNOT6L, CNTN5, CPT1A, CRLF1, CRTAC1, CTSK, CTTN, CUL5, CWC15, DCAF12, DENND4C, DHX16, DLAT, DLG2* *(2x), DNAH6, DNAJA1, DNAJB13, DNAJC1 (4x), DOCK5, DSCAML1 (2x), EGFR, EHBP1L1, ELF2, ENAM, EPYC, ETV6 (2x), FAF1, FAM13A, FAM161A, FBXL18, FCHSD2 (3x), FOCAD (2x), FOXO4, FOXP1, FXYD2 (2x), FXYD6, GNAQ, GRIA4 (2x), GRIP1, GTDC1, HELQ (2x), HIPK3, HIVEP3, HK1, HSD17B3, IER2, IFT46, IKZF1, INTS12, invKMT2A(i9-e18), ITGAL, JAM2, JAML, KCNIP4, KDM2A, KIAA0586, KIF27, KIF5B (2x), KLF3, KLHL13, LINC02550, LMO2, LOC390877, LOC441179, LPXN, LRBA, MACF1, MALAT1, MAN1A1, MCL1, MDM1, MED1, MEF2A, MEF2C, MMP13, MPZL2, MPZL3 (2x), MSRB3, MYO5B, NAV2, NBPF19, NCAM1, NDUFA6-DT, NDUFS3, NNMT, NRG3, NT5C2, NWD2, OSBPL9, OXSR1, PACS1, PARP14, PBRM1, PBX1, PDE10A, PDE1A, PDE6C, PHLDB1, PITPNA, PIWIL4, PKHD1, PLRG1, PPM1G, PPP6R3, PRCP, RAP1GAP2, RASSF4, RBM4, RDH5, RELA, RIN2, RNF115, RNF25, RORA, RPS3, RRM2B, RUNX1, SCGB1D1, SCN3B, SCN4B (2x), SEC14L1, SEC16A, SETDB1, SIK3 (2x), SMIM35, SNAPC3, SORCS2, SORL1 (3x), SRSF4, SUN1, SVIL (2x), TACC1, TAF3, TCF12, TIMM44, TLN1, TMEM123, TMEM135, TNRC6B, TNRC6C, TNXB, TPTE2P5, TRAPPC4, TTF1, TTLL11, TTLL5, TUBGCP2, UBASH3B (2x), UBE4A (3x), UGT8, UNC93A, UNC93B1, USP2-AS1, USP20, UTRN, UVRAG, WDTC1, WNT5B, ZBTB5, ZC3H12C, ZC3HAV1, ZEB1, ZNF57* and *ZYG11B.*
